# Supplementary material for: Ratiometric Near-Infrared Fluorescent Probes Based on Hemicyanine Dyes Bearing Dithioacetal and Formal Residues for pH Detection in Mitochondria
Source: Molecules. 2021 Apr 6;26(7):2088. doi: 10.3390/molecules26072088 (PMC8038704; doi:10.3390/molecules26072088)
Supplement: Supplementary file 1 [file molecules-26-02088-s001.pdf]

Ratiometric Near-infrared Fluorescent Probes Based on Hemicyanine Dyes Bearing Dithioacetal and Formal Residues for pH Detection in Mitochondria

Yunnan Yan,[ab] Shuai Xia,[a] Yibin Zhang,[a*] Shulin Wan,[a] Tara Vohs,[a] Marina Tanasova[a], Rudy L. Luck,[a*] and Haiying Liu[a*]

[a]Department of Chemistry, Department of Biological Sciences, Michigan Technological University, Houghton, MI 49931, USA. E-mail : yibinz@mtu.edu ; rluck@mtu.edu ; mtanasov@mtu.edu ; hyliu@mtu.edu

[b]College of Pharmaceutical Sciences, Gannan Medical University, Ganzhou, Jiangxi 341000, China.

Index

Figure S1. GaussView representation of Probe **A.** 4

Figure S2. Calculated UV-Vis spectrum for Probe **A** in water. 4

Table S1. Calculated atomic coordinates for Probe **A** in water. 5

Table S2. Excitation energies and oscillator strengths listing for Probe **A** in water. 6

Figure S3. Drawings of selected molecular orbitals for probe **A** listed in Table S2 as excited state 1. 6

Figure S4. GaussView representation of Probe **AH$^+$.** 7

Figure S5. Calculated UV-Vis spectrum for Probe **AH$^+$** in water. 7

Table S3. Calculated atomic coordinates for Probe **AH$^+$** in water. 8

Table S4. Excitation energies and oscillator strengths listing for Probe **AH$^+$** in water. 9

Figure S6. Drawings of selected molecular orbitals for probe **AH$^+$** listed in Table S4 as excited state 1. 10

Figure S7. GaussView representation of Probe **B**. 10

Table S5. Calculated atomic coordinates for Probe **B** in water. 11

Figure S8. Calculated UV-Vis spectrum for Probe B in water 12

Table S6. Excitation energies and oscillator strengths listing for Probe **B** in water. 12

Figure S9. Drawings of selected molecular orbitals for probe **B** listed in Table S6 as excited state 1. 13

Figure S10. GaussView representation of Probe **BH$^+$**. 13

Figure S11. Calculated UV-Vis spectrum for Probe **BH^{++}** in water 14

Table S7. Calculated atomic coordinates for Probe **BH$^+$** in water. 14

Table S8. Excitation energies and oscillator strengths listing for Probe **BH$^+$** in water. 15

Figure S12. Drawings of selected molecular orbitals for probe **BH$^+$** listed in Table S8 as excited state 1. 16

Table S9. Converged atomic positions for probe **A** with the SMD method. 17

Table S10. Converged atomic positions for probe **AH$^+$** with the SMD method... 18

Table S11. Converged atomic positions for probe **B** with the SMD method.. 19

Table S12. Converged atomic positions for probe **BH$^+$** with the SMD method. 20

Table S13. Converged atomic positions for probe **A** with the SMD$_{Bondi}$ method. 21

Table S14. Converged atomic positions for probe **AH$^+$** with the SMD$_{Bondi}$ method. 22

Table S15. Converged atomic positions for probe **B** with the SMD$_{Bondi}$ method...................................... 23

Table S16. Converged atomic positions for probe **BH$^+$** with the SMD$_{Bondi}$ method. 24

Table S17. Converged atomic positions for probe **A** with the SMD$_{SAS}$ method... 25

Table S18. Converged atomic positions for probe **AH$^+$** with the SMD$_{SAS}$ method. 26

Table S19. Converged atomic positions for probe **B** with the SMD$_{SAS}$ method... 27

Table S20. Converged atomic positions for probe **BH$^+$** with the SMD$_{SAS}$ method. 28

Table S21. Converged atomic positions for probe **A-**H_2O with the SMD method. 29

Table S22. Converged atomic positions for probe **AH$^+$-**H_2O with the SMD method. 30

Table S23. Converged atomic positions for probe **B-**H_2O with the SMD method. 31

Table S24. Converged atomic positions for probe **BH$^+$-**H_2O with the SMD method. 32

Table S25. Converged atomic positions for probe **A**-H_2O with the SMD$_{Bondi}$ method. 33

Table S26. Converged atomic positions for probe **AH$^+$**-H_2O with the SMD$_{Bondi}$ method. 34

Table S27. Converged atomic positions for probe **B**-H_2O with the SMD$_{Bondi}$ method. 35

Table S28. Converged atomic positions for probe **BH$^+$**-H_2O with the SMD$_{Bondi}$ method. 36

Table S29. Converged atomic positions for probe **A**-H_2O with the SMD$_{SAS}$ method. 37

Table S30. Converged atomic positions for probe **AH$^+$**-H_2O with the SMD$_{SAS}$ method. 38

Table S31. Converged atomic positions for probe **B**-H_2O with the SMD$_{SAS}$ method. 39

Table S32. Converged atomic positions for probe **BH$^+$**-H_2O with the SMD$_{SAS}$ method. 40

Figure S13. Drawing of the model with the intramolecular H···S bond preserved. 41

Table S33. Converged atomic positions for probe **A**-H_2O with the intramolecular H···S bond preserved and with the SMD method... 42

Table S34. Converged atomic positions for probe **A**-H_2O with the intramolecular H···S bond preserved and with the SMD$_{Bondi}$ method... 43

Table S35. Converged atomic positions for probe **A**-H_2O with the intramolecular H···S bond preserved and with the SMD_{SAS} method. 44

Figure S14 ^{1}H NMR spectrum of of probe **AH^{++}** in $CDCl_3$ solution 46

Figure S15 ^{13}C NMR spectrum of of probe **AH^{++}** in $CDCl_3$ Solutions... 47

Figure S16 High-resolution mass spectrum of of probe **AH^{++}** 48

Figure S17 ^{1}H NMR spectrum of of probe **BH^{++}** in $CDCl_3$ solutions 49

Figure S18 ^{13}CNMR spectrum of of probe **BH^{++}** in $CDCl_3$ solutions 50

Figure S19 High-resolution mass spectrum of of probe **AH^{++}** 51

Table S36 Reversible pH cyclic data of probe **AH^{++}** **52**

Table S37 Reversible pH cyclic data of probe **AH^{++}** **53**

Table S38. Fluorescence intensities of 10 µM probes **AH^{++}** in the absence and presence of different cations (200 µM) in pH 7.4 buffers under excitation at 635 nm...54

Table S39. Fluorescence intensities of 10 µM probes **BH^{++}** in the absence and presence of different cations (200 µM) in pH 7.4 buffers under excitation at 635 nm...55

Table S40. Fluorescence intensities of 10 µM probes **AH^{++}** in the absence and presence of different anions (200 µM) in pH 7.4 buffers under excitation at 635 nm..56

Table S41. Fluorescence intensities of 10 µM probes **BH^{++}** in the absence and presence of different anions (200 µM) in pH 7.4 buffers under excitation at 635 nm...57

Table S42. Fluorescence intensities of 10 µM probes **AH^{++}** in the absence and presence of different Amino acids (200 µM) in pH 7.4 buffers under excitation at 635 nm..58

Table S43. Fluorescence intensities of 10 µM probes **BH^{++}** in the absence and presence of different Amino acids (200 µM) in pH 7.4 buffers under excitation at 635 nm..59

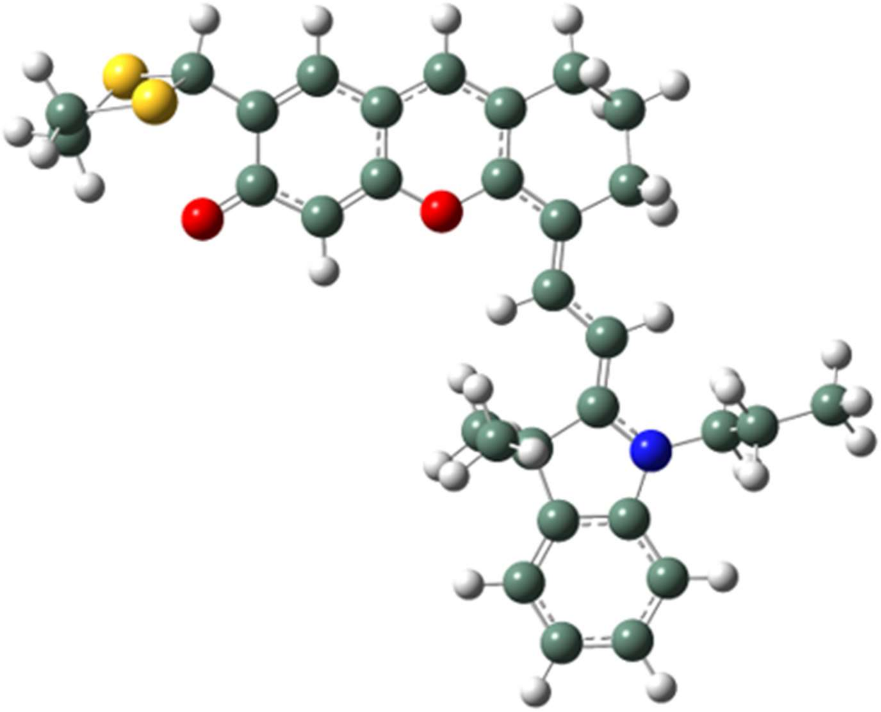

Figure S1. GaussView representation of Probe A.

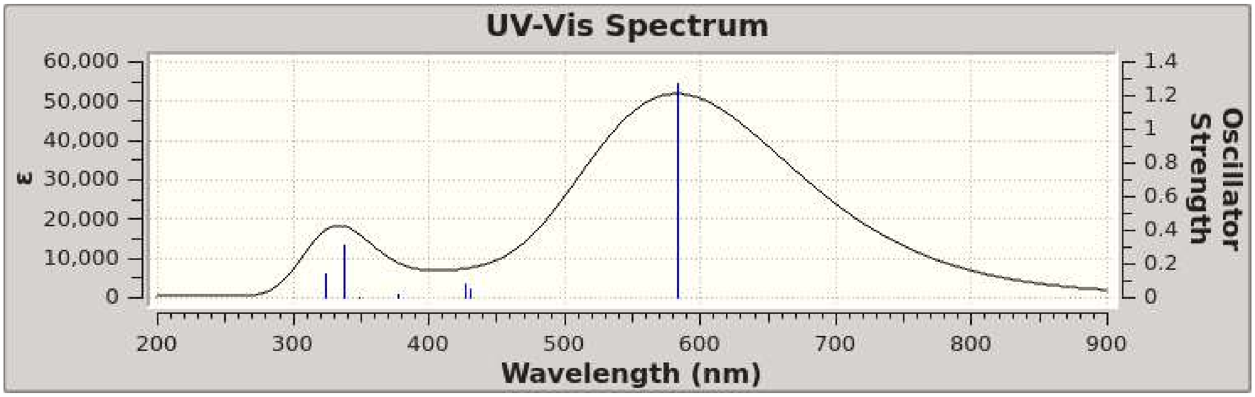

Figure S2. Calculated UV-Vis spectrum for Probe A in water.

Table S1. Calculated atomic coordinates for Probe A in water.

Row	Symbol	X	Y	Z	Row	Symbol	X	Y	Z
1	C	-4.94475	0.153054	-0.00525	36	C	7.14285	-1.95508	-0.10281
2	C	-4.02111	-0.98275	-0.13304	37	H	-1.90771	-1.47363	-0.28445
3	C	-2.62247	-0.66144	-0.19248	38	H	-5.16306	2.27035	0.124058
4	C	-2.19286	0.634806	-0.14358	39	H	-3.20948	3.87311	0.088806
5	C	-3.0865	1.735236	-0.03066	40	H	2.032947	3.833811	0.799803
6	C	-4.46963	1.436175	0.034664	41	H	2.622128	3.556869	-0.82622
7	O	-0.85281	0.871282	-0.21286	42	H	1.224204	5.628868	-0.65931
8	C	-0.32942	2.11737	-0.16253	43	H	0.575713	4.46847	-1.81449
9	C	-1.17181	3.230316	-0.0544	44	H	-1.267	5.335549	-0.39769
10	C	-2.54222	3.017613	0.006357	45	H	-0.39403	4.883309	1.056241
11	C	1.087026	2.195504	-0.21203	46	H	1.28414	0.107479	-0.20946
12	C	1.711982	3.56994	-0.21801	47	H	3.792097	1.904823	-0.20874
13	C	0.762168	4.640709	-0.74715	48	H	-6.91216	0.918729	0.098575
14	C	-0.56159	4.602812	0.006964	49	H	-6.53244	-3.06641	-0.37807
15	C	1.837295	1.037996	-0.21781	50	H	-8.22219	-3.09693	-0.97465
16	C	3.240554	0.970697	-0.21694	51	H	-9.00487	-1.81964	0.951999
17	C	3.969626	-0.19975	-0.20524	52	H	-7.92436	-3.05026	1.642193
18	O	-4.42844	-2.16368	-0.1939	53	H	2.418926	-2.97297	1.200019
19	C	-6.4233	-0.0568	0.095461	54	H	1.714283	-1.35499	1.150453
20	S	-7.22064	-0.96116	-1.29808	55	H	3.229944	-1.63062	2.019275
21	C	-7.47697	-2.52625	-0.41381	56	H	1.722757	-1.45607	-1.47506
22	S	-6.87707	-0.90627	1.678548	57	H	3.241599	-1.80962	-2.31034
23	C	-7.9751	-2.18345	0.976989	58	H	2.420265	-3.07624	-1.38695
24	C	3.459511	-1.63899	-0.14815	59	H	7.112647	0.672758	-0.76294
25	C	4.753125	-2.4153	-0.10932	60	H	5.713645	1.70881	-0.88388
26	C	5.823	-1.52501	-0.14792	61	H	5.521099	1.719308	1.638585
27	N	5.328866	-0.22124	-0.23266	62	H	6.947737	0.701509	1.737401
28	C	2.654808	-1.90771	1.132418	63	H	7.561685	3.109638	2.090221
29	C	2.660651	-2.00992	-1.40675	64	H	8.318597	2.506605	0.61394
30	C	6.185017	0.948722	-0.25586	65	H	6.881869	3.529845	0.516083
31	C	6.470474	1.488453	1.142	66	H	4.155917	-4.48082	-0.01261
32	C	7.356086	2.725427	1.087642	67	H	6.503865	-5.30172	0.053294
33	C	4.982883	-3.77631	-0.04022	68	H	8.386052	-3.70019	0.005963
34	C	6.303952	-4.23593	-0.00282	69	H	7.977738	-1.26246	-0.11376
35	C	7.364856	-3.33193	-0.03123					

Table S2. Excitation energies and oscillator strengths listing for Probe A in water.

Excited State	Nature	E (eV)	λ(nm)	*f*	Orbital transitions	Normalized coefficient
1:	A	2.1241	583.69	1.2685	137 ->138	0.70634
2:	A	2.8766	431.01	0.0517	135 ->138	0.49859
					136 ->138	0.46847
3:	A	2.9029	427.10	0.0772	135 ->138	-0.47144
					136 ->138	0.48850
4:	A	3.2818	377.79	0.0199	134 ->138	0.68430
					136 ->138	0.10496
					137 ->139	0.12233
5:	A	3.5545	348.81	0.0006	133 ->138	0.67965
					135 ->138	0.14214
6:	A	3.6734	337.52	0.3055	130 ->138	0.16080
					131 ->138	0.13172
					132 ->138	0.23435
					134 ->138	-0.10749
					137 ->139	0.60928
7:	A	3.8284	323.85	0.1448	132 ->138	0.63872
					136 ->138	0.11989
					137 ->139	-0.23591

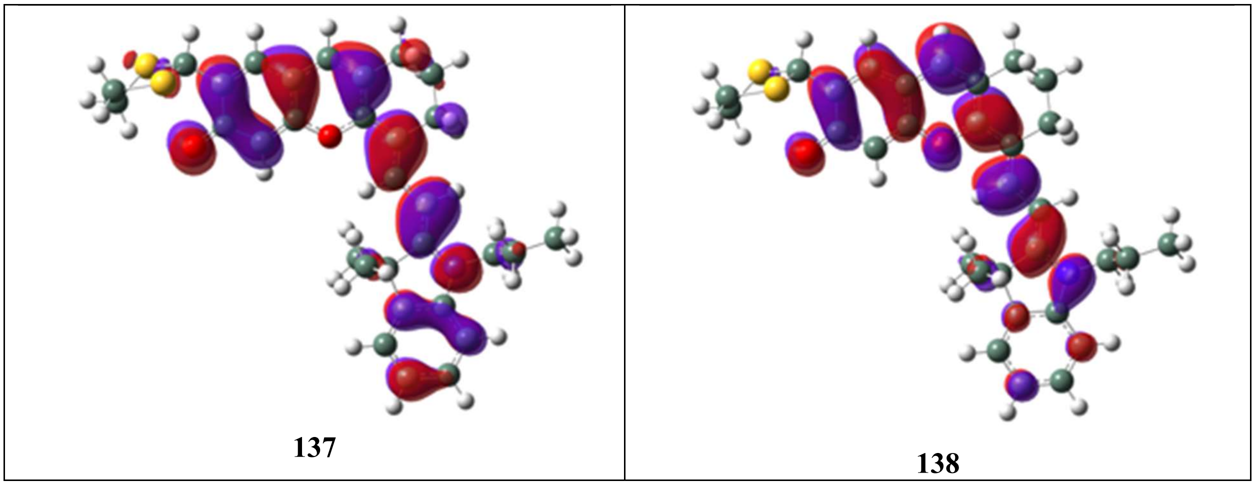

Figure S3. Drawings of selected molecular orbitals for probe A listed in Table S2 as excited state 1.

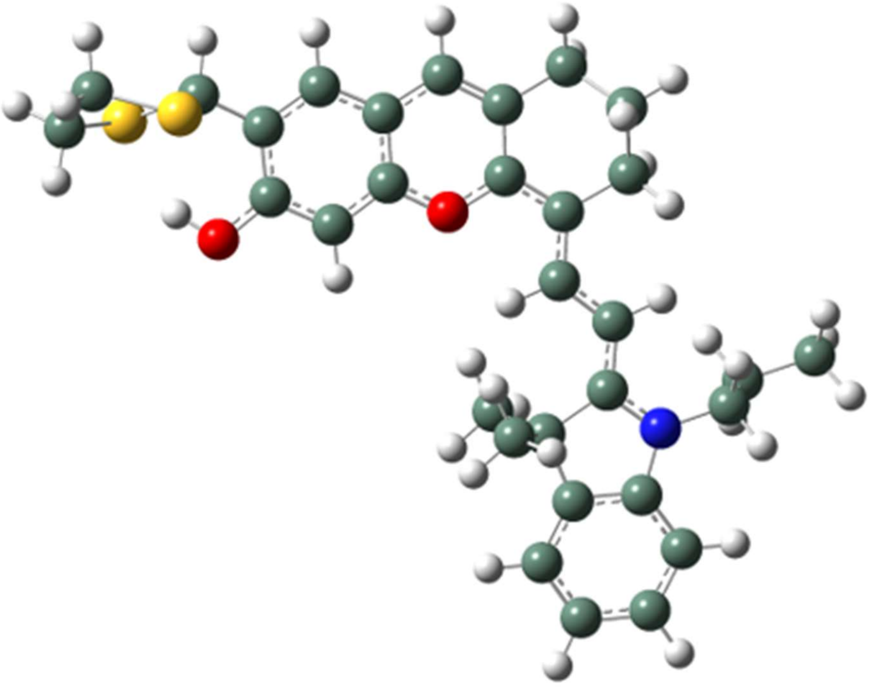

Figure S4. GaussView representation of Probe AH^{++}.

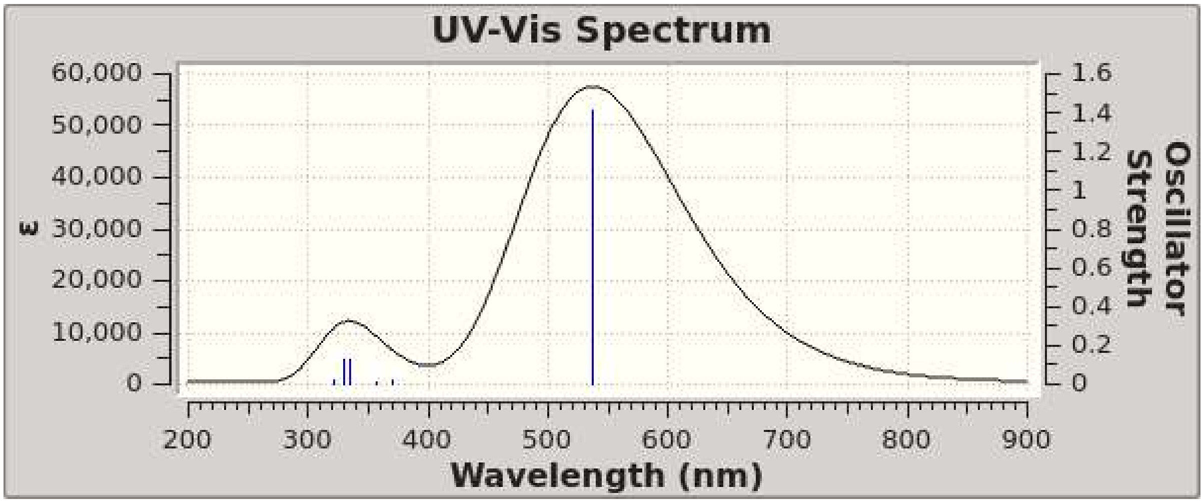

Figure S5. Calculated UV-Vis spectrum for Probe AH^{++} in water.

Table S3. Calculated atomic coordinates for Probe AH^{++} in water.

Row	Symbol	X	Y	Z	Row	Symbol	X	Y	Z
1	C	-4.8635	0.074175	-0.25398	36	C	7.19066	-1.91481	0.204633
2	C	-3.91755	-0.9831	-0.16371	37	H	-1.84334	-1.51907	0.016878
3	C	-2.55906	-0.70741	-0.05121	38	H	-5.11142	2.197473	-0.33918
4	C	-2.14014	0.609062	-0.04141	39	H	-3.17405	3.843841	-0.25415
5	C	-3.03808	1.681748	-0.15617	40	H	2.581306	3.52382	0.942887
6	C	-4.40122	1.378608	-0.26323	41	H	2.134284	3.832687	-0.72242
7	O	-0.80955	0.835872	0.075769	42	H	1.201882	5.609843	0.671813
8	C	-0.28649	2.083806	0.068803	43	H	0.489213	4.44605	1.784021
9	C	-1.15428	3.211655	-0.06016	44	H	-1.25698	5.323587	0.224367
10	C	-2.49726	2.998657	-0.15912	45	H	-0.28775	4.818716	-1.1513
11	C	1.101509	2.184066	0.178996	46	H	1.331119	0.083303	0.139525
12	C	1.726561	3.555365	0.259526	47	H	3.800916	1.912726	0.265759
13	C	0.741049	4.619617	0.730786	48	H	-5.18199	-2.35747	-0.53696
14	C	-0.5307	4.576359	-0.10768	49	H	-6.85657	0.761733	-0.5282
15	C	1.873925	1.01822	0.189064	50	H	-7.75872	-3.20911	-0.018
16	C	3.25743	0.974993	0.236906	51	H	-9.04504	-2.46245	-0.9885
17	C	4.004798	-0.20296	0.226758	52	H	-8.98181	-1.85222	1.581389
18	O	-4.27513	-2.27668	-0.1702	53	H	-9.12184	-0.49849	0.443809
19	C	-6.34469	-0.16202	-0.25616	54	H	1.725936	-1.48875	1.40586
20	S	-6.91251	-1.4635	-1.46761	55	H	2.443465	-3.09976	1.320065
21	C	-8.1587	-2.26209	-0.38234	56	H	3.220618	-1.83764	2.286385
22	S	-6.91654	-0.6651	1.406596	57	H	1.799587	-1.35726	-1.2178
23	C	-8.48096	-1.3222	0.767993	58	H	3.345993	-1.59807	-2.04249
24	C	3.506942	-1.64116	0.132715	59	H	2.529168	-2.96317	-1.26835
25	C	4.806147	-2.40516	0.126145	60	H	5.696621	1.726532	0.968612
26	C	5.864269	-1.50755	0.210467	61	H	7.101383	0.694182	0.902217
27	N	5.346164	-0.20557	0.2924	62	H	7.027877	0.723092	-1.60819
28	C	2.671221	-2.03186	1.362268	63	H	5.603706	1.748211	-1.55745
29	C	2.747104	-1.89621	-1.17883	64	H	7.664653	3.127161	-1.9321
30	C	6.196794	0.972596	0.357959	65	H	6.933004	3.548123	-0.38153
31	C	6.533729	1.510913	-1.02848	66	H	8.36737	2.517262	-0.4321
32	C	7.42318	2.742885	-0.93781	67	H	4.238557	-4.47832	-0.01629
33	C	5.053887	-3.76326	0.047184	68	H	6.596999	-5.26409	-0.01571
34	C	6.381048	-4.20195	0.047141	69	H	8.456735	-3.64347	0.113965
35	C	7.43123	-3.28669	0.122091	70	H	8.015029	-1.21179	0.253199

Table S4. Excitation energies and oscillator strengths listing for Probe AH^{+} in water.

Excited State	Nature	E (eV)	λ (nm)	*f*	Orbital transitions	Normalized coefficient
1:	A	2.3051	537.87	1.4119	137 ->138	0.70674
2:	A	3.3517	369.91	0.0143	134 ->138	-0.11180
					135 ->138	-0.37967
					136 ->138	0.56762
3:	A	3.3547	369.58	0.0242	133 ->138	-0.10437
					134 ->138	0.20711
					135 ->138	0.50425
					136 ->138	0.41581
4:	A	3.4777	356.51	0.0094	133 ->138	-0.13008
					134 ->138	0.63161
					135 ->138	-0.23721
					137 ->139	0.14231
5:	A	3.6944	335.60	0.1288	131 ->138	-0.10638
					133 ->138	-0.39019
					134 ->138	-0.18289
					137 ->139	0.52913
					137 ->140	0.10244
6:	A	3.7531	330.35	0.1208	132 ->138	-0.13513
					133 ->138	0.54022
					135 ->138	0.14837
					137 ->139	0.38654
7:	A	3.8498	322.05	0.0224	132 ->138	0.67875

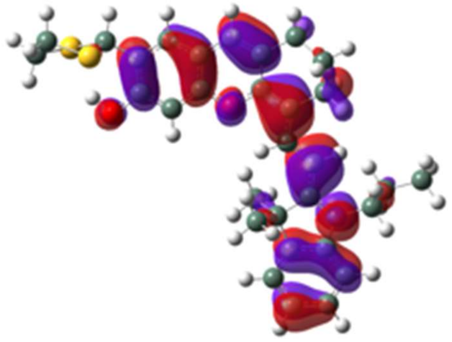 137	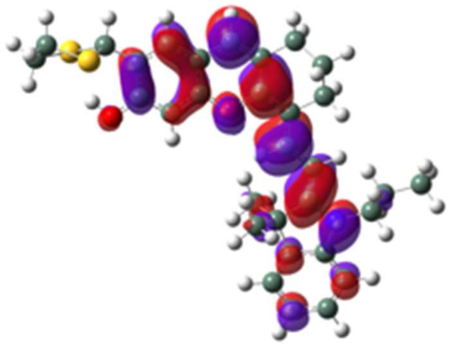 138

Figure S6. Drawings of selected molecular orbitals for probe AH^{+} listed in Table S4 as excited state 1.

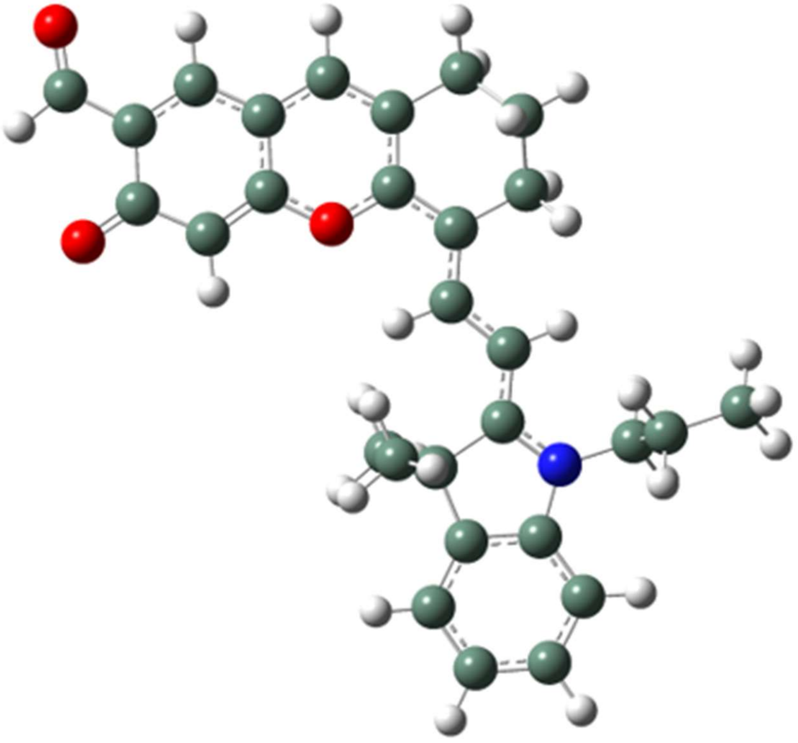

Figure S7. GaussView representation of Probe B.

Table S5. Calculated atomic coordinates for Probe B in water.

Row	Symbol	X	Y	Z	Row	Symbol	X	Y	Z
1	C	-6.03115	-1.35456	0.11349	32	C	5.842781	-3.47848	-0.00658
2	C	-4.90872	-2.29129	0.23142	33	C	6.702141	-2.38746	-0.13244
3	C	-3.5965	-1.70086	0.187799	34	C	6.209643	-1.08565	-0.23884
4	C	-3.44021	-0.35245	0.046141	35	H	-2.72816	-2.34711	0.271476
5	C	-4.5366	0.554425	-0.06652	36	H	-6.68553	0.662158	-0.11042
6	C	-5.82316	0.004688	-0.02692	37	H	-5.07314	2.625886	-0.3
7	O	-2.16823	0.138705	0.017702	38	H	0.728222	3.46074	0.346738
8	C	-1.89858	1.457089	-0.12724	39	H	0.072155	3.536655	-1.27566
9	C	-2.95312	2.388957	-0.24872	40	H	-1.03743	5.228119	0.098456
10	C	-4.24807	1.922582	-0.21062	41	H	-1.4204	4.04598	1.346282
11	C	-0.53588	1.809918	-0.15854	42	H	-3.43173	4.460806	-0.06119
12	C	-0.1784	3.274565	-0.23797	43	H	-2.52036	4.058983	-1.50745
13	C	-1.29977	4.178835	0.264078	44	H	0.068003	-0.20562	-0.08662
14	C	-2.61118	3.840442	-0.43439	45	H	2.166588	2.040563	-0.29675
15	C	0.430391	0.812627	-0.145	46	H	0.845051	-1.48479	1.322758
16	C	1.807902	1.020034	-0.22073	47	H	1.849466	-2.9354	1.38918
17	C	2.756266	0.008503	-0.209	48	H	2.429394	-1.42566	2.107357
18	O	-5.07429	-3.52575	0.362813	49	H	0.732114	-1.71866	-1.2911
19	C	-7.38915	-1.8765	0.144745	50	H	2.243441	-1.80478	-2.20695
20	O	-8.40825	-1.2029	0.054457	51	H	1.741938	-3.16312	-1.18957
21	H	-7.46518	-2.97132	0.259347	52	H	5.630191	1.452544	-0.96559
22	C	2.540359	-1.49571	-0.06882	53	H	4.051332	2.188428	-1.0591
23	C	3.960363	-2.00388	-0.08124	54	H	3.960638	2.27359	1.471059
24	C	4.830697	-0.92666	-0.21621	55	H	5.566427	1.567449	1.535814
25	N	4.081328	0.252315	-0.3141	56	H	5.698024	4.060997	1.769038
26	C	1.871881	-1.84937	1.268582	57	H	6.493612	3.558028	0.275696
27	C	1.763769	-2.07281	-1.26258	58	H	4.876292	4.267142	0.220091
28	C	4.688219	1.5671	-0.42536	59	H	3.789487	-4.1406	0.123773
29	C	4.915725	2.214762	0.936787	60	H	6.252465	-4.48072	0.074287
30	C	5.529335	3.600319	0.792231	61	H	7.776138	-2.54793	-0.14604
31	C	4.457371	-3.2898	0.02107	62	H	6.889705	-0.2453	-0.32778

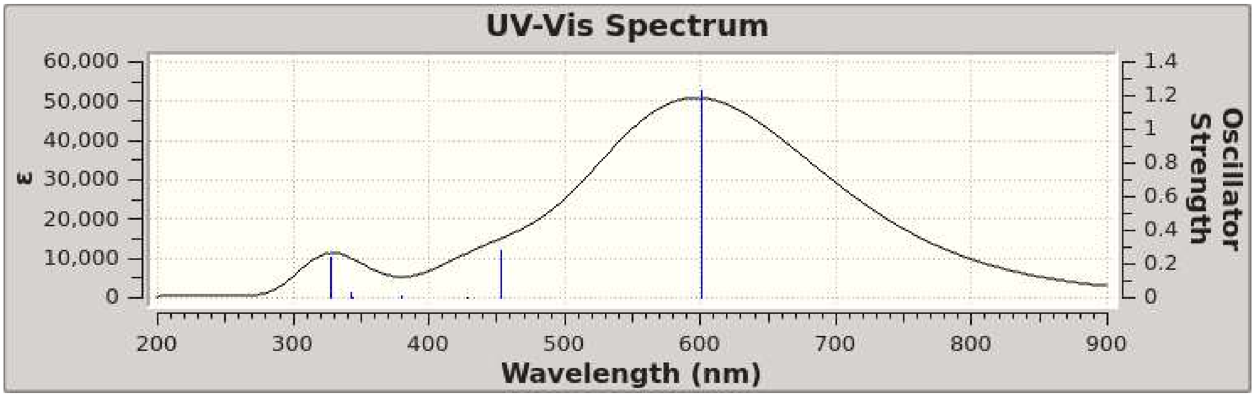

Figure S8. Calculated UV-Vis spectrum for Probe B in water.

Table S6. Excitation energies and oscillator strengths listing for Probe B in water.

Excited State	Nature	E (eV)	(nm)	*f*	Orbital transitions	Normalized coefficient
1:	A	2.0653	600.32	1.2288	117 ->118	0.70211
2:	A	2.7395	452.58	0.2841	116 ->118	0.68716
3:	A	2.8933	428.51	0.0000	115 ->118	0.68194
					115 ->119	-0.14906
4:	A	3.2641	379.84	0.0116	116 ->119	0.11661
					117 ->119	0.68317
					117 ->120	-0.10497
5:	A	3.6102	343.43	0.0001	111 ->118	0.44264
					111 ->119	-0.19102
					112 ->118	0.19503
					115 ->118	-0.14010
					115 ->119	-0.41064
					115 ->120	0.13884
6:	A	3.6206	342.44	0.0348	112 ->118	-0.13550
					114 ->118	0.61085
					117 ->120	-0.30839
7:	A	3.7888	327.24	0.2385	114 ->118	0.30097
					116 ->119	0.20791
					117 ->120	0.57861

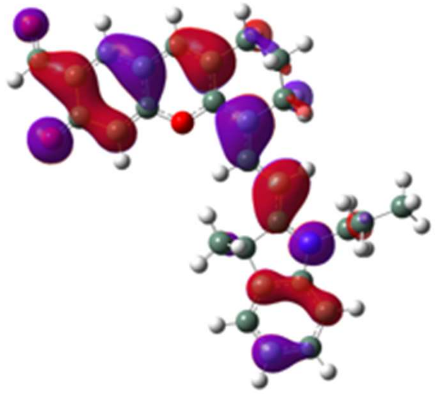	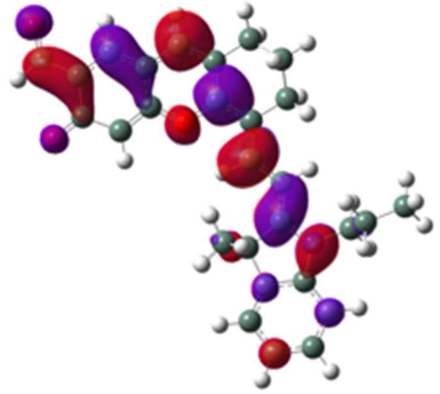
**117**	**118**

Figure S9. Drawings of selected molecular orbitals for probe B listed in Table S6 as excited state 1.

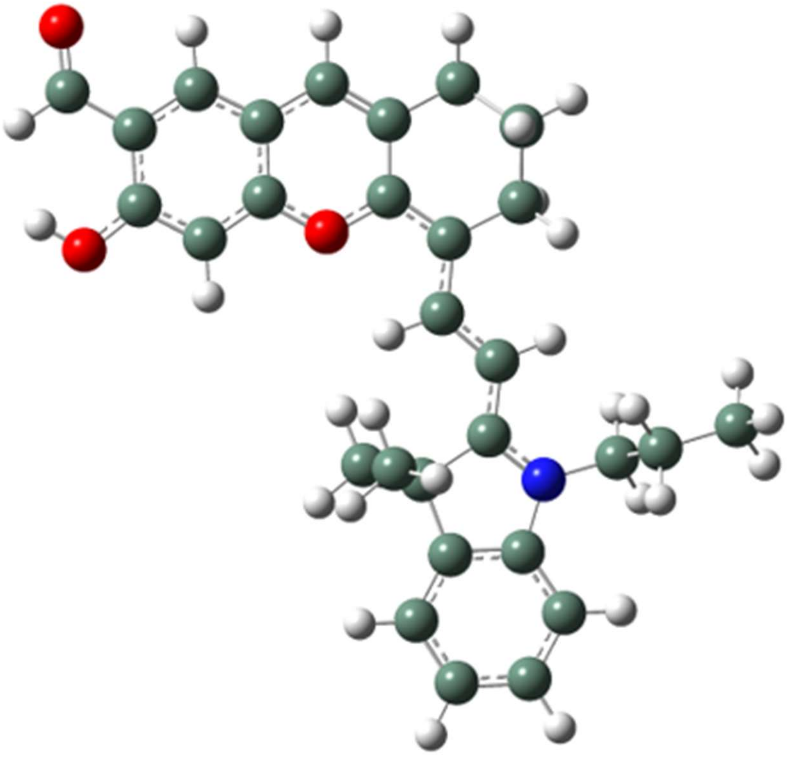

Figure S10. GaussView representation of Probe BH^{+}.

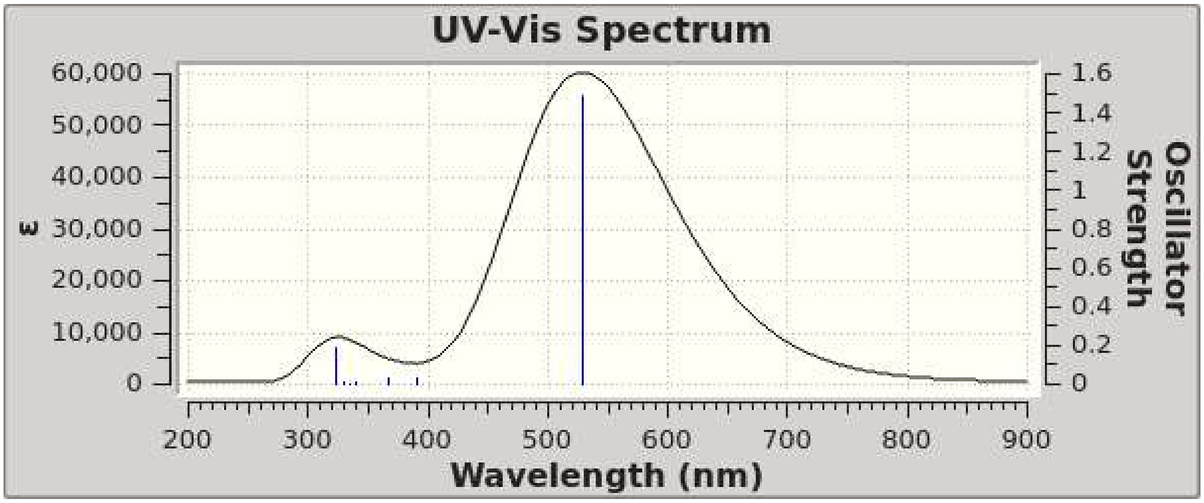

Figure S11. Calculated UV-Vis spectrum for Probe BH^+ in water.

Table S7. Calculated atomic coordinates for Probe BH^+ in water.

Row	Symbol	X	Y	Z	Row	Symbol	X	Y	Z
1	C	-5.99277	-1.33015	0.127156	25	N	4.085193	0.250666	-0.3034
2	C	-4.86852	-2.19166	0.211216	26	C	1.87974	-1.85107	1.258325
3	C	-3.57981	-1.66801	0.158114	27	C	1.774377	-2.06473	-1.27758
4	C	-3.41552	-0.30427	0.023541	28	C	4.698099	1.568016	-0.40086
5	C	-4.50565	0.586104	-0.06652	29	C	4.925235	2.192753	0.971796
6	C	-5.78262	0.042009	-0.01151	30	C	5.55457	3.573101	0.846615
7	O	-2.14779	0.158926	-0.01516	31	C	4.465985	-3.29409	0.004148
8	C	-1.86252	1.480497	-0.14806	32	C	5.850947	-3.47787	-0.02071
9	C	-2.93307	2.425898	-0.26934	33	C	6.711522	-2.38565	-0.13618
10	C	-4.21527	1.977155	-0.21703	34	C	6.219206	-1.08432	-0.235
11	C	-0.5185	1.830274	-0.16963	35	H	-2.72062	-2.32579	0.224139
12	C	-0.14425	3.290822	-0.22928	36	H	-6.65077	0.690652	-0.07785
13	C	-1.26978	4.203549	0.245873	37	H	-5.04144	2.677861	-0.30224
14	C	-2.56895	3.866878	-0.47593	38	H	0.744979	3.461435	0.386004
15	C	0.456179	0.818215	-0.15433	39	H	0.143129	3.553852	-1.25665
16	C	1.817881	1.028031	-0.22317	40	H	-1.0002	5.249753	0.075815
17	C	2.772682	0.004156	-0.21057	41	H	-1.41068	4.080951	1.326497
18	O	-4.93867	-3.52692	0.343804	42	H	-3.39242	4.505019	-0.14367
19	C	-7.36903	-1.82738	0.180284	43	H	-2.4456	4.048775	-1.55228
20	O	-8.35524	-1.12186	0.107417	44	H	0.090493	-0.19876	-0.09955
21	H	-7.51361	-2.91964	0.295827	45	H	2.180624	2.047128	-0.29173
22	C	2.551149	-1.49693	-0.07864	46	H	-5.84411	-3.85166	0.378998
23	C	3.968795	-2.00706	-0.09032	47	H	0.853048	-1.48651	1.31114
24	C	4.840356	-0.93138	-0.21404	48	H	1.856902	-2.93717	1.374452

Row	Symbol	X	Y	Z	Row	Symbol	X	Y	Z
49	H	2.436333	-1.43036	2.098965	57	H	5.721589	4.018444	1.830612
50	H	0.742305	-1.71235	-1.30313	58	H	6.521508	3.52638	0.335931
51	H	2.253757	-1.79076	-2.22013	59	H	4.911991	4.253918	0.279436
52	H	1.754315	-3.15502	-1.20996	60	H	3.799961	-4.14693	0.098187
53	H	5.638916	1.452281	-0.9414	61	H	6.262096	-4.47989	0.053729
54	H	4.064671	2.19785	-1.02798	62	H	7.785197	-2.54639	-0.14807
55	H	3.968034	2.255559	1.501732	63	H	6.898373	-0.24288	-0.31717
56	H	5.565798	1.530974	1.565449					

Table S8. Excitation energies and oscillator strengths listing for Probe BH^+ in water.

Excited State	Nature	E (eV)	(nm)	*f*	Orbital transitions	Normalized coefficient
1:	A	2.3417	529.47	1.4804	117 ->118	0.70646
2:	A	3.1700	391.12	0.0332	116 ->118	-0.20204
					117 ->119	0.66519
3:	A	3.3711	367.79	0.0285	115 ->118	0.11677
					116 ->118	0.64243
					117 ->119	0.19618
					117 ->120	-0.15657
4:	A	3.6478	339.89	0.0100	115 ->118	0.67859
					116 ->118	-0.10974
					117 ->120	0.10472
5:	A	3.7002	335.08	0.0001	113 ->118	0.50554
					113 ->119	-0.47726
6:	A	3.7509	330.54	0.0099	114 ->118	0.68197
					117 ->120	0.13082
7:	A	3.8465	322.33	0.1886	114 ->118	-0.11022
					115 ->119	-0.13553
					116 ->118	0.14335
					116 ->119	-0.13618
					117 ->120	0.63410

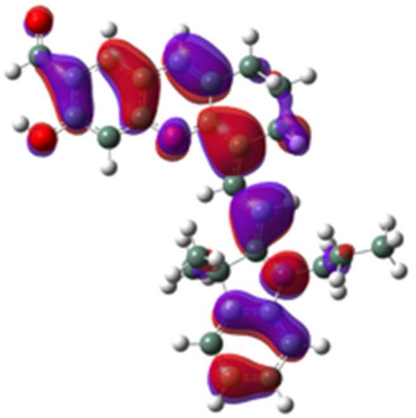

117

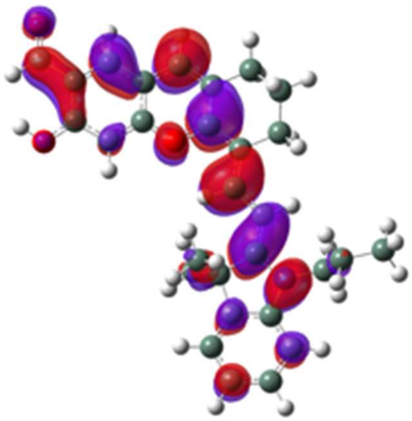

118

Figure S12. Drawings of selected molecular orbitals for probe BH^+ listed in Table S8 as excited state 1.

Table S9. Converged atomic positions for probe A with the SMD method.

Row	Symbol	X	Y	Z	Row	Symbol	X	Y	Z
1	C	-4.87719	0.023904	0.025468	35	C	7.446403	-3.40277	-0.01461
2	C	-3.94923	-1.1027	-0.06106	36	C	7.219123	-2.02713	-0.09265
3	C	-2.55653	-0.7824	-0.10729	37	H	-1.83598	-1.59134	-0.17531
4	C	-2.13026	0.518957	-0.07213	38	H	-5.10231	2.143631	0.117207
5	C	-3.02425	1.613871	0.011661	39	H	-3.15531	3.757111	0.110567
6	C	-4.40379	1.312305	0.054705	40	H	2.076227	3.735676	0.879727
7	O	-0.78632	0.752869	-0.12759	41	H	2.678887	3.450485	-0.74264
8	C	-0.26473	2.004218	-0.08368	42	H	1.271671	5.52241	-0.58989
9	C	-1.11824	3.118812	0.009345	43	H	0.630807	4.352632	-1.74436
10	C	-2.48281	2.904899	0.047456	44	H	-1.2208	5.219881	-0.32858
11	C	1.145404	2.091285	-0.13172	45	H	-0.3406	4.76331	1.124802
12	C	1.76575	3.467887	-0.1399	46	H	1.362772	-0.00167	-0.13169
13	C	0.814317	4.532472	-0.67789	47	H	3.854218	1.818594	-0.16122
14	C	-0.51055	4.491577	0.073953	48	H	-6.83407	0.809996	0.060056
15	C	1.906399	0.934482	-0.14335	49	H	-6.57066	-3.21945	-0.27075
16	C	3.305267	0.883433	-0.15826	50	H	-8.2421	-3.18596	-0.90709
17	C	4.040613	-0.28831	-0.15679	51	H	-9.01041	-1.80859	0.948806
18	O	-4.34547	-2.30324	-0.1023	52	H	-8.0001	-3.06106	1.709519
19	C	-6.36026	-0.17207	0.099048	53	H	2.532119	-3.0576	1.285229
20	S	-7.13935	-1.11015	-1.28392	54	H	1.804151	-1.44568	1.22458
21	C	-7.48335	-2.6297	-0.3495	55	H	3.335076	-1.69336	2.08447
22	S	-6.86136	-0.96385	1.69727	56	H	1.765509	-1.57455	-1.36298
23	C	-7.99889	-2.2163	1.014173	57	H	3.272635	-1.89429	-2.24136
24	C	3.535504	-1.72687	-0.08469	58	H	2.501875	-3.18207	-1.2966
25	C	4.830922	-2.49771	-0.06045	59	H	7.172265	0.603452	-0.74159
26	C	5.896695	-1.60475	-0.12042	60	H	5.768975	1.647715	-0.81183
27	N	5.39525	-0.30244	-0.20878	61	H	5.619562	1.59918	1.702095
28	C	2.750785	-1.98895	1.209082	62	H	7.044865	0.565926	1.755001
29	C	2.716015	-2.11017	-1.32559	63	H	7.68229	2.961298	2.154386
30	C	6.252493	0.870411	-0.21609	64	H	8.405069	2.383727	0.647931
31	C	6.561117	1.370838	1.189867	65	H	6.973055	3.420399	0.601326
32	C	7.454825	2.602	1.146764	66	H	4.237929	-4.56046	0.059379
33	C	5.06494	-3.8578	0.014584	67	H	6.594382	-5.37479	0.096407
34	C	6.388686	-4.31073	0.03598	68	H	8.468724	-3.76763	0.009641
					69	H	8.046947	-1.32747	-0.12367

Table S10. Converged atomic positions for probe AH^+ with the SMD method.

Row	Symbol	X	Y	Z
1	C	-4.90801	0.257876	-0.14502
2	C	-3.98692	-0.81076	-0.02063
3	C	-2.61955	-0.57168	0.055419
4	C	-2.16576	0.734046	0.010067
5	C	-3.0384	1.823289	-0.11326
6	C	-4.41126	1.548955	-0.19057
7	O	-0.82417	0.927016	0.094922
8	C	-0.27227	2.166452	0.051283
9	C	-1.11853	3.309514	-0.08856
10	C	-2.46775	3.12721	-0.15859
11	C	1.118215	2.238482	0.141448
12	C	1.772047	3.597676	0.194096
13	C	0.813138	4.690581	0.653886
14	C	-0.46534	4.657582	-0.17486
15	C	1.869299	1.057065	0.154972
16	C	3.251241	0.995185	0.21416
17	C	3.979839	-0.19529	0.189979
18	O	-4.47473	-2.06391	0.013397
19	C	-6.3941	0.063728	-0.21808
20	S	-7.00742	-1.00162	-1.59488
21	C	-7.54019	-2.4066	-0.57015
22	S	-7.06123	-0.58818	1.378945
23	C	-8.18711	-1.83894	0.676653
24	C	3.464539	-1.61891	0.021093
25	C	4.751951	-2.4003	0.006587
26	C	5.819982	-1.52423	0.161429
27	N	5.318447	-0.22057	0.296411
28	C	2.595256	-2.05871	1.209266
29	C	2.728361	-1.79737	-1.31507
30	C	6.186523	0.941739	0.417975
31	C	6.578745	1.507263	-0.94134
32	C	7.488689	2.716752	-0.78283
33	C	4.980553	-3.75682	-0.13286
34	C	6.301371	-4.21513	-0.12184
35	C	7.362463	-3.32061	0.025243
36	C	7.140388	-1.95045	0.169939
37	H	-1.91659	-1.39369	0.148183
38	H	-5.1006	2.382576	-0.28991
39	H	-3.12682	3.985099	-0.26018
40	H	2.632602	3.554923	0.869121
41	H	2.172741	3.848316	-0.79765
42	H	1.293938	5.669373	0.57101
43	H	0.564043	4.538649	1.711136
44	H	-1.17563	5.425028	0.144317
45	H	-0.22082	4.864382	-1.22561
46	H	1.311047	0.131121	0.102877
47	H	3.811446	1.921657	0.268389
48	H	-3.75068	-2.69766	0.094371
49	H	-6.84829	1.03841	-0.39891
50	H	-6.68594	-3.03931	-0.32858
51	H	-8.26043	-2.97611	-1.16391
52	H	-8.32079	-2.61027	1.44079
53	H	-9.15653	-1.38849	0.451717
54	H	1.647849	-1.51794	1.23996
55	H	2.376362	-3.12537	1.112058
56	H	3.118964	-1.89818	2.155149
57	H	1.792183	-1.23686	-1.33702
58	H	3.351684	-1.46599	-2.1496
59	H	2.497697	-2.85672	-1.45502
60	H	5.675056	1.690514	1.025483
61	H	7.069915	0.632432	0.980299
62	H	7.079663	0.724274	-1.52167
63	H	5.669892	1.780035	-1.48938
64	H	7.77583	3.123775	-1.75637
65	H	6.991418	3.514048	-0.22055
66	H	8.406661	2.453403	-0.24699
67	H	4.15337	-4.4502	-0.25151
68	H	6.504454	-5.27564	-0.2323
69	H	8.382134	-3.69294	0.0256
70	H	7.969064	-1.25962	0.277059

Table S11. Converged atomic positions for probe B with the SMD method.

Row	Symbol	X	Y	Z
1	C	-6.04444	-1.3438	0.16504
2	C	-4.92749	-2.26901	0.322412
3	C	-3.61834	-1.69267	0.267066
4	C	-3.45622	-0.34727	0.081377
5	C	-4.54522	0.556659	-0.06749
6	C	-5.8283	0.016726	-0.02099
7	O	-2.1787	0.131641	0.047191
8	C	-1.90084	1.449987	-0.13708
9	C	-2.96068	2.384058	-0.29491
10	C	-4.25094	1.928234	-0.25255
11	C	-0.54683	1.800407	-0.17109
12	C	-0.18138	3.261744	-0.27161
13	C	-1.30875	4.18034	0.187712
14	C	-2.60645	3.824628	-0.52729
15	C	0.426308	0.79819	-0.14579
16	C	1.794372	1.018651	-0.21934
17	C	2.75294	0.007913	-0.21713
18	O	-5.09036	-3.51421	0.499975
19	C	-7.39583	-1.85864	0.20438
20	O	-8.41803	-1.18075	0.077695
21	H	-7.48625	-2.94534	0.360227
22	C	2.54751	-1.49739	-0.09987
23	C	3.970203	-1.99295	-0.11443
24	C	4.831592	-0.90764	-0.23098
25	N	4.069822	0.266562	-0.31749
26	C	1.877039	-1.8777	1.229098
27	C	1.77922	-2.06174	-1.30444
28	C	4.674295	1.588862	-0.3829
29	C	4.914183	2.177623	1.001717
30	C	5.545932	3.558594	0.901741
31	C	4.476806	-3.27648	-0.02791
32	C	5.864084	-3.44997	-0.05311
33	C	6.714844	-2.34915	-0.16116
34	C	6.211869	-1.05033	-0.25183
35	H	-2.75178	-2.33646	0.377616
36	H	-6.68263	0.678864	-0.13398
37	H	-5.07452	2.627987	-0.37003
38	H	0.714278	3.449588	0.32903
39	H	0.090227	3.499083	-1.30954
40	H	-1.04173	5.223857	-0.00258
41	H	-1.45053	4.072864	1.270068
42	H	-3.43499	4.458089	-0.19822
43	H	-2.48375	3.994896	-1.60563
44	H	0.067756	-0.2211	-0.08379
45	H	2.15072	2.040216	-0.28508
46	H	0.839888	-1.54063	1.270979
47	H	1.886354	-2.96588	1.33348
48	H	2.418078	-1.44506	2.074643
49	H	0.744589	-1.71462	-1.3219
50	H	2.259672	-1.77131	-2.24234
51	H	1.773108	-3.15323	-1.24269
52	H	5.612853	1.491515	-0.93214
53	H	4.027723	2.235158	-0.97957
54	H	3.959239	2.231279	1.536669
55	H	5.561489	1.499538	1.569239
56	H	5.720998	3.984783	1.893527
57	H	6.508631	3.51654	0.381481
58	H	4.899246	4.249345	0.350489
59	H	3.811883	-4.13044	0.060518
60	H	6.283439	-4.44873	0.015638
61	H	7.789696	-2.50098	-0.17323
62	H	6.87894	-0.19921	-0.32966

Table S12. Converged atomic positions for probe BH^{+} with the SMD method.

Row	Symbol	X	Y	Z
1	C	-5.99475	-1.32171	0.229038
2	C	-4.87353	-2.18284	0.310721
3	C	-3.583	-1.66874	0.233929
4	C	-3.4217	-0.30418	0.079344
5	C	-4.50989	0.586582	-0.00977
6	C	-5.78927	0.047194	0.068319
7	O	-2.15129	0.155063	0.021071
8	C	-1.86558	1.479929	-0.13141
9	C	-2.93885	2.422249	-0.25309
10	C	-4.2206	1.975924	-0.18131
11	C	-0.52314	1.828851	-0.16949
12	C	-0.15071	3.288844	-0.24973
13	C	-1.27239	4.205231	0.22706
14	C	-2.577	3.859906	-0.48077
15	C	0.454556	0.816882	-0.1532
16	C	1.813559	1.034973	-0.22726
17	C	2.771465	0.012426	-0.21901
18	O	-5.10084	-3.50155	0.464133
19	C	-7.35214	-1.85381	0.309808
20	O	-8.3599	-1.16262	0.240557
21	H	-7.4437	-2.9427	0.440876
22	C	2.555669	-1.48883	-0.08944
23	C	3.974635	-1.99218	-0.09536
24	C	4.841521	-0.91365	-0.22171
25	N	4.08203	0.264606	-0.31904
26	C	1.877765	-1.85331	1.24059
27	C	1.789506	-2.05692	-1.29439
28	C	4.697579	1.582799	-0.40357
29	C	4.959987	2.17624	0.97469
30	C	5.616323	3.544262	0.857206
31	C	4.475594	-3.27727	0.005322
32	C	5.861751	-3.45448	-0.01577
33	C	6.718131	-2.35849	-0.13443
34	C	6.221018	-1.05922	-0.23997
35	H	-2.71607	-2.31845	0.296309
36	H	-6.65081	0.704243	0.003171
37	H	-5.04795	2.674888	-0.26504
38	H	0.746679	3.462331	0.352424
39	H	0.120647	3.538138	-1.28463
40	H	-1.0056	5.249603	0.042778
41	H	-1.40501	4.089199	1.309491
42	H	-3.40104	4.497518	-0.14994
43	H	-2.45695	4.024115	-1.56023
44	H	0.09215	-0.20093	-0.09399
45	H	2.17611	2.054136	-0.29307
46	H	-4.26615	-3.98657	0.49934
47	H	0.841476	-1.51293	1.274288
48	H	1.883927	-2.94039	1.354236
49	H	2.416619	-1.41443	2.084187
50	H	0.757034	-1.70432	-1.31972
51	H	2.276729	-1.77663	-2.23176
52	H	1.77781	-3.14762	-1.22308
53	H	5.627669	1.469519	-0.96357
54	H	4.049366	2.229594	-0.9969
55	H	4.010498	2.251496	1.516634
56	H	5.599776	1.490798	1.541655
57	H	5.805551	3.975462	1.84414
58	H	6.574613	3.479244	0.3314
59	H	4.978792	4.241809	0.304002
60	H	3.807627	-4.12784	0.101422
61	H	6.277555	-4.45387	0.064314
62	H	7.792126	-2.51554	-0.14324
63	H	6.890775	-0.21117	-0.32628

Table S13. Converged atomic positions for probe A with the SMD_{Bondi} method.

Row	Symbol	X	Y	Z	Row	Symbol	X	Y	Z
1	C	-4.88131	0.029053	0.047261	35	C	7.454999	-3.39588	-0.054
2	C	-3.95366	-1.10199	0.004058	36	C	7.223186	-2.02061	-0.11859
3	C	-2.55902	-0.7837	-0.02933	37	H	-1.83898	-1.59448	-0.06963
4	C	-2.13333	0.517432	-0.01885	38	H	-5.10671	2.150421	0.090641
5	C	-3.02833	1.615399	0.031049	39	H	-3.15982	3.758065	0.085384
6	C	-4.40938	1.31682	0.058816	40	H	2.069441	3.75257	0.884313
7	O	-0.78932	0.75149	-0.06581	41	H	2.676886	3.437814	-0.73035
8	C	-0.26893	2.003339	-0.04938	42	H	1.270275	5.510301	-0.62276
9	C	-1.12136	3.118733	0.0155	43	H	0.634539	4.319036	-1.7563
10	C	-2.48709	2.905043	0.046366	44	H	-1.22262	5.212488	-0.36848
11	C	1.142402	2.088752	-0.09932	45	H	-0.35094	4.788699	1.098491
12	C	1.76217	3.46504	-0.13072	46	H	1.356428	-0.00191	-0.07643
13	C	0.813108	4.519062	-0.6928	47	H	3.849689	1.814937	-0.13247
14	C	-0.51501	4.49292	0.053408	48	H	-6.84845	0.798586	0.018441
15	C	1.90216	0.93262	-0.10093	49	H	-6.5849	-3.24307	-0.25762
16	C	3.30165	0.879509	-0.12485	50	H	-8.23104	-3.19135	-0.95549
17	C	4.039483	-0.2892	-0.13385	51	H	-9.04223	-1.77307	0.854743
18	O	-4.35717	-2.29731	-0.01327	52	H	-8.08537	-3.03274	1.669331
19	C	-6.36354	-0.17597	0.082592	53	H	2.545824	-3.07549	1.295736
20	S	-7.08459	-1.13892	-1.31886	54	H	1.817374	-1.46534	1.260312
21	C	-7.48521	-2.64061	-0.37652	55	H	3.355114	-1.72029	2.101037
22	S	-6.90712	-0.96078	1.670343	56	H	1.771103	-1.55519	-1.35118
23	C	-8.0427	-2.20015	0.961765	57	H	3.274305	-1.89164	-2.22559
24	C	3.539112	-1.7298	-0.06924	58	H	2.487102	-3.17101	-1.2851
25	C	4.837152	-2.49741	-0.06231	59	H	7.156742	0.614361	-0.7611
26	C	5.899733	-1.60087	-0.1271	60	H	5.751068	1.653005	-0.80083
27	N	5.394142	-0.29968	-0.19885	61	H	5.653742	1.603096	1.716798
28	C	2.764386	-2.00655	1.227246	62	H	7.076784	0.568285	1.739886
29	C	2.714881	-2.10203	-1.31055	63	H	7.724451	2.961611	2.129095
30	C	6.247022	0.875337	-0.21588	64	H	8.419054	2.38429	0.6103
31	C	6.58354	1.373866	1.184508	65	H	6.988916	3.423391	0.590127
32	C	7.478224	2.603356	1.125833	66	H	4.252673	-4.56336	0.047488
33	C	5.076056	-3.85708	-0.00069	67	H	6.60965	-5.37037	0.052272
34	C	6.400782	-4.30675	0.002084	68	H	8.478252	-3.75793	-0.0446
					69	H	8.05013	-1.32056	-0.15334

Table S14. Converged atomic positions for probe AH^+ with the SMD_{Bondi} method.

Row	Symbol	X	Y	Z
1	C	-4.90116	0.258849	-0.16351
2	C	-3.97995	-0.8105	-0.05094
3	C	-2.61331	-0.57228	0.041407
4	C	-2.16273	0.735872	0.023492
5	C	-3.03648	1.825394	-0.08582
6	C	-4.40782	1.551047	-0.17975
7	O	-0.82278	0.930686	0.120856
8	C	-0.27337	2.171299	0.100369
9	C	-1.12106	3.31533	-0.01904
10	C	-2.46888	3.131015	-0.10052
11	C	1.117144	2.244044	0.189098
12	C	1.768721	3.603133	0.264407
13	C	0.809486	4.68461	0.749345
14	C	-0.4713	4.666605	-0.07561
15	C	1.87003	1.064386	0.181656
16	C	3.252462	1.003138	0.219323
17	C	3.98331	-0.18526	0.192736
18	O	-4.47876	-2.06161	-0.04503
19	C	-6.38499	0.057913	-0.25506
20	S	-6.97291	-0.98562	-1.66064
21	C	-7.53589	-2.40369	-0.67042
22	S	-7.06561	-0.63086	1.320059
23	C	-8.19563	-1.85309	0.577029
24	C	3.465203	-1.61349	0.079418
25	C	4.752888	-2.39475	0.055683
26	C	5.823393	-1.51367	0.153693
27	N	5.324183	-0.20662	0.259501
28	C	2.629388	-2.01188	1.305588
29	C	2.695855	-1.83748	-1.23117
30	C	6.195636	0.957309	0.324246
31	C	6.560218	1.483084	-1.05872
32	C	7.489513	2.683403	-0.95393
33	C	4.979826	-3.75453	-0.04793
34	C	6.300681	-4.21161	-0.05818
35	C	7.3638	-3.3128	0.032784
36	C	7.14383	-1.93927	0.140526
37	H	-1.90504	-1.38968	0.126226
38	H	-5.09688	2.385542	-0.26778
39	H	-3.12954	3.988603	-0.18941
40	H	2.631099	3.550453	0.936241
41	H	2.165829	3.874199	-0.72321
42	H	1.288222	5.665584	0.685932
43	H	0.564513	4.511033	1.80398
44	H	-1.18169	5.425038	0.263515
45	H	-0.23157	4.899392	-1.12176
46	H	1.312257	0.138498	0.126805
47	H	3.811867	1.930476	0.259032
48	H	-3.76743	-2.7059	0.048409
49	H	-6.84637	1.030973	-0.42186
50	H	-6.69191	-3.04963	-0.42793
51	H	-8.2511	-2.953	-1.28784
52	H	-8.34996	-2.63477	1.325646
53	H	-9.15499	-1.38505	0.346714
54	H	1.69214	-1.45522	1.35794
55	H	2.390093	-3.07648	1.243523
56	H	3.186586	-1.83727	2.229225
57	H	1.753997	-1.28723	-1.25088
58	H	3.294492	-1.52792	-2.09129
59	H	2.469248	-2.90164	-1.33453
60	H	5.702462	1.725114	0.922581
61	H	7.090258	0.662484	0.876046
62	H	7.036125	0.680193	-1.63238
63	H	5.64279	1.754595	-1.59235
64	H	7.753425	3.064235	-1.94417
65	H	7.019722	3.500385	-0.39679
66	H	8.419228	2.420482	-0.43907
67	H	4.151637	-4.45238	-0.1228
68	H	6.501874	-5.27459	-0.14142
69	H	8.38333	-3.68424	0.017207
70	H	7.975524	-1.24706	0.202511

Table S15. Converged atomic positions for probe B with the SMD_{Bondi} method.

Row	Symbol	X	Y	Z
1	C	-6.03554	-1.36217	0.094981
2	C	-4.91517	-2.29204	0.219089
3	C	-3.60649	-1.70657	0.184601
4	C	-3.44962	-0.35625	0.043313
5	C	-4.54256	0.548819	-0.07721
6	C	-5.82523	0.001781	-0.04611
7	O	-2.17422	0.130633	0.024585
8	C	-1.90278	1.453573	-0.12205
9	C	-2.96336	2.386751	-0.25097
10	C	-4.25349	1.923212	-0.22055
11	C	-0.54705	1.808635	-0.14847
12	C	-0.18841	3.272583	-0.22615
13	C	-1.31268	4.17595	0.269542
14	C	-2.61795	3.836264	-0.43907
15	C	0.426432	0.810925	-0.13371
16	C	1.79673	1.028305	-0.21123
17	C	2.749422	0.014731	-0.20522
18	O	-5.0772	-3.53786	0.350657
19	C	-7.38733	-1.88305	0.120357
20	O	-8.41026	-1.20517	0.028908
21	H	-7.46751	-2.97672	0.231532
22	C	2.534815	-1.4879	-0.06012
23	C	3.95444	-1.99354	-0.07777
24	C	4.821992	-0.91632	-0.22113
25	N	4.068702	0.261239	-0.3222
26	C	1.87437	-1.83965	1.281387
27	C	1.752958	-2.0717	-1.24676
28	C	4.679688	1.576809	-0.42743
29	C	4.955576	2.200131	0.935532
30	C	5.585516	3.576989	0.784137
31	C	4.45335	-3.27844	0.027196
32	C	5.838874	-3.46266	-0.00675
33	C	6.695805	-2.37035	-0.14141
34	C	6.200758	-1.07022	-0.25064
35	H	-2.73788	-2.35041	0.274233
36	H	-6.68371	0.661891	-0.13652
37	H	-5.07915	2.623443	-0.31526
38	H	0.7154	3.453774	0.363704
39	H	0.065249	3.532996	-1.26298
40	H	-1.05049	5.224753	0.104217
41	H	-1.44097	4.040301	1.350233
42	H	-3.44348	4.455278	-0.07695
43	H	-2.51247	4.045063	-1.51225
44	H	0.069142	-0.20889	-0.07624
45	H	2.153558	2.049174	-0.28451
46	H	0.843559	-1.48544	1.334117
47	H	1.866249	-2.92591	1.402295
48	H	2.431614	-1.40502	2.114895
49	H	0.717209	-1.72794	-1.26231
50	H	2.222067	-1.79771	-2.1949
51	H	1.746209	-3.16187	-1.16839
52	H	5.6048	1.464204	-0.99612
53	H	4.025565	2.212752	-1.02684
54	H	4.01632	2.269092	1.49533
55	H	5.616724	1.536981	1.504181
56	H	5.789542	4.026669	1.759583
57	H	6.532944	3.521252	0.238202
58	H	4.924648	4.255873	0.235458
59	H	3.784879	-4.12689	0.136261
60	H	6.251639	-4.46272	0.075925
61	H	7.769198	-2.52942	-0.15981
62	H	6.873899	-0.22626	-0.34719

Table S16. Converged atomic positions for probe BH^+ with the SMD_{Bondi} method.

Row	Symbol	X	Y	Z
1	C	-5.99339	-1.33898	0.139285
2	C	-4.87141	-2.19976	0.199469
3	C	-3.58119	-1.68126	0.153992
4	C	-3.42207	-0.3115	0.047723
5	C	-4.51148	0.578835	-0.02047
6	C	-5.79032	0.033892	0.028726
7	O	-2.15269	0.152724	0.015365
8	C	-1.8695	1.48097	-0.09821
9	C	-2.94332	2.424638	-0.19572
10	C	-4.22415	1.972884	-0.14438
11	C	-0.52705	1.832925	-0.1236
12	C	-0.15753	3.294528	-0.17424
13	C	-1.27736	4.196449	0.333031
14	C	-2.58523	3.870047	-0.37723
15	C	0.451669	0.823757	-0.11968
16	C	1.810605	1.040567	-0.20135
17	C	2.765649	0.015973	-0.19212
18	O	-5.10215	-3.52371	0.302306
19	C	-7.35322	-1.87464	0.189923
20	O	-8.35822	-1.18134	0.144986
21	H	-7.44307	-2.96869	0.273807
22	C	2.544537	-1.48148	-0.02428
23	C	3.961037	-1.99233	-0.04332
24	C	4.830499	-0.92162	-0.21051
25	N	4.075575	0.258294	-0.32022
26	C	1.888655	-1.8099	1.325752
27	C	1.755321	-2.07795	-1.20066
28	C	4.694967	1.569072	-0.46342
29	C	5.017958	2.206941	0.882201
30	C	5.669263	3.568605	0.689385
31	C	4.458014	-3.27708	0.076889
32	C	5.84231	-3.46219	0.033233
33	C	6.701326	-2.3742	-0.12668
34	C	6.208384	-1.07552	-0.25191
35	H	-2.71136	-2.32742	0.200955
36	H	-6.65463	0.688206	-0.02178
37	H	-5.05212	2.672257	-0.20985
38	H	0.743847	3.457093	0.424439
39	H	0.104687	3.569107	-1.20494
40	H	-1.01252	5.245632	0.17674
41	H	-1.40366	4.051128	1.412457
42	H	-3.40748	4.495448	-0.02028
43	H	-2.47363	4.071037	-1.45111
44	H	0.090957	-0.1948	-0.06529
45	H	2.172371	2.059345	-0.27576
46	H	-4.2692	-4.0097	0.326997
47	H	0.859614	-1.45074	1.377845
48	H	1.877168	-2.89426	1.460656
49	H	2.451748	-1.36627	2.150363
50	H	0.720123	-1.73317	-1.21597
51	H	2.220902	-1.81777	-2.15425
52	H	1.748082	-3.16661	-1.10628
53	H	5.600312	1.435965	-1.05843
54	H	4.028758	2.201501	-1.05183
55	H	4.095004	2.303439	1.464394
56	H	5.681548	1.54173	1.445198
57	H	5.905059	4.030971	1.651556
58	H	6.60176	3.484793	0.122038
59	H	5.007625	4.249846	0.144765
60	H	3.789636	-4.12277	0.204491
61	H	6.254535	-4.46139	0.127762
62	H	7.773873	-2.5367	-0.15262
63	H	6.881315	-0.23397	-0.36802

Table S17. Converged atomic positions for probe A with the SMD$_{SAS}$ method.

Row	Symbol	X	Y	Z
1	C	-4.85105	0.002546	0.05468
2	C	-3.91959	-1.11497	0.030661
3	C	-2.533	-0.79778	0.009401
4	C	-2.11024	0.508503	0.010905
5	C	-3.00754	1.599221	0.041297
6	C	-4.38433	1.29631	0.059174
7	O	-0.76792	0.742957	-0.02709
8	C	-0.24832	1.994542	-0.02446
9	C	-1.10836	3.113366	0.026449
10	C	-2.4682	2.897306	0.04803
11	C	1.158389	2.085022	-0.08163
12	C	1.776667	3.462036	-0.1265
13	C	0.824895	4.511107	-0.69114
14	C	-0.50103	4.486552	0.05809
15	C	1.924106	0.928043	-0.08718
16	C	3.319357	0.875651	-0.12636
17	C	4.059239	-0.29637	-0.14363
18	O	-4.31763	-2.32889	0.020568
19	C	-6.33261	-0.20772	0.079308
20	S	-7.03487	-1.17551	-1.33719
21	C	-7.57446	-2.63172	-0.38938
22	S	-6.88288	-1.00467	1.656496
23	C	-8.11375	-2.13968	0.93795
24	C	3.559104	-1.73409	-0.06803
25	C	4.854214	-2.50235	-0.07493
26	C	5.917395	-1.60663	-0.14759
27	N	5.409767	-0.30517	-0.22089
28	C	2.804023	-2.00099	1.240358
29	C	2.714531	-2.11067	-1.29191
30	C	6.256007	0.875075	-0.2296
31	C	6.540373	1.391037	1.174578
32	C	7.394136	2.64877	1.134345
33	C	5.091667	-3.86227	-0.01762
34	C	6.415828	-4.31405	-0.02494
35	C	7.471419	-3.40449	-0.08563
36	C	7.240267	-2.02879	-0.14612
37	H	-1.80922	-1.60585	-0.01636
38	H	-5.08762	2.1248	0.077369
39	H	-3.14471	3.747511	0.073299
40	H	2.088365	3.758044	0.884915
41	H	2.68958	3.430684	-0.72894
42	H	1.2802	5.503958	-0.62747
43	H	0.644059	4.304708	-1.75315
44	H	-1.20941	5.206849	-0.3611
45	H	-0.33125	4.782142	1.102396
46	H	1.383514	-0.00834	-0.05538
47	H	3.869468	1.809565	-0.13997
48	H	-6.82093	0.763999	0.013282
49	H	-6.73934	-3.32003	-0.25285
50	H	-8.35445	-3.12145	-0.9773
51	H	-9.07005	-1.62746	0.813291
52	H	-8.23922	-2.96178	1.646839
53	H	2.585224	-3.06927	1.318737
54	H	1.860551	-1.45442	1.279857
55	H	3.408169	-1.71033	2.103651
56	H	1.772113	-1.5613	-1.31561
57	H	3.258601	-1.9039	-2.21716
58	H	2.488318	-3.17987	-1.25782
59	H	7.184199	0.617283	-0.74227
60	H	5.771718	1.643585	-0.83594
61	H	5.588563	1.592047	1.67868
62	H	7.044273	0.604279	1.747384
63	H	7.605889	3.018299	2.141698
64	H	8.353112	2.46056	0.640348
65	H	6.889845	3.450264	0.584462
66	H	4.266611	-4.56589	0.035984
67	H	6.623569	-5.37813	0.020892
68	H	8.494173	-3.76802	-0.08342
69	H	8.066123	-1.32815	-0.18654

Table S18. Converged atomic positions for probe AH^+ with the SMDSAS method.

Row	Symbol	X	Y	Z
1	C	-4.89513	0.239778	-0.13395
2	C	-3.96602	-0.82815	-0.11354
3	C	-2.59766	-0.58515	-0.0807
4	C	-2.15284	0.725265	-0.06148
5	C	-3.03437	1.813478	-0.0845
6	C	-4.40717	1.535243	-0.12112
7	O	-0.81129	0.926432	-0.01475
8	C	-0.26862	2.170474	0.005762
9	C	-1.1255	3.313644	-0.04275
10	C	-2.47422	3.12296	-0.07348
11	C	1.123116	2.246508	0.0859
12	C	1.768054	3.608143	0.178191
13	C	0.818021	4.670683	0.718731
14	C	-0.48552	4.670358	-0.06906
15	C	1.880482	1.06902	0.080615
16	C	3.2617	1.004141	0.151147
17	C	3.992452	-0.18546	0.162243
18	O	-4.45423	-2.08869	-0.13326
19	C	-6.38157	0.042637	-0.15573
20	S	-7.04711	-0.93405	-1.58035
21	C	-7.63334	-2.36954	-0.62761
22	S	-6.98544	-0.72247	1.415309
23	C	-8.20953	-1.84944	0.672797
24	C	3.477618	-1.61229	0.034796
25	C	4.762267	-2.3953	0.060812
26	C	5.830307	-1.51503	0.192854
27	N	5.3296	-0.20779	0.282028
28	C	2.594334	-2.00878	1.22546
29	C	2.75685	-1.83499	-1.3009
30	C	6.193833	0.959515	0.367191
31	C	6.582316	1.486497	-1.00686
32	C	7.488148	2.702233	-0.88796
33	C	4.989291	-3.75598	-0.02574
34	C	6.308706	-4.2174	0.012924
35	C	7.370331	-3.32031	0.134415
36	C	7.149233	-1.94553	0.225908
37	H	-1.88386	-1.40138	-0.0657
38	H	-5.10291	2.368397	-0.13965
39	H	-3.14282	3.978472	-0.09863
40	H	2.652193	3.545755	0.819795
41	H	2.132025	3.909091	-0.81381
42	H	1.290604	5.656029	0.670722
43	H	0.60312	4.464812	1.774305
44	H	-1.18973	5.412662	0.316006
45	H	-0.27564	4.941151	-1.11262
46	H	1.32695	0.142119	0.015044
47	H	3.823576	1.929249	0.201199
48	H	-3.72872	-2.72286	-0.10359
49	H	-6.85008	1.021168	-0.25149
50	H	-6.81363	-3.06787	-0.45407
51	H	-8.40021	-2.85745	-1.23374
52	H	-8.37031	-2.65767	1.390309
53	H	-9.15277	-1.32454	0.508673
54	H	1.658844	-1.44784	1.237941
55	H	2.357117	-3.07349	1.153876
56	H	3.11463	-1.83546	2.170734
57	H	1.818866	-1.2799	-1.34947
58	H	3.38653	-1.52555	-2.13898
59	H	2.533368	-2.89897	-1.4138
60	H	5.684158	1.727154	0.951919
61	H	7.07981	0.67389	0.936322
62	H	7.084379	0.689326	-1.56626
63	H	5.671399	1.740095	-1.56004
64	H	7.77077	3.083678	-1.87314
65	H	6.991401	3.513765	-0.34619
66	H	8.409179	2.45809	-0.34847
67	H	4.161189	-4.4506	-0.12566
68	H	6.50977	-5.28151	-0.05563
69	H	8.388952	-3.69409	0.156569
70	H	7.977926	-1.25305	0.315176

Table S19. Converged atomic positions for probe B with the SMD_{SAS} method.

Row	Symbol	X	Y	Z
1	C	-6.04954	-1.35536	0.041135
2	C	-4.93265	-2.28852	0.039121
3	C	-3.62637	-1.71402	-0.00888
4	C	-3.46054	-0.35502	-0.05217
5	C	-4.5502	0.557819	-0.05967
6	C	-5.83076	0.019097	-0.00869
7	O	-2.1842	0.11898	-0.08045
8	C	-1.90336	1.447993	-0.13607
9	C	-2.96627	2.395516	-0.17447
10	C	-4.25422	1.942506	-0.12243
11	C	-0.5507	1.798887	-0.15703
12	C	-0.18467	3.263271	-0.17087
13	C	-1.28967	4.149739	0.392853
14	C	-2.61504	3.850778	-0.29502
15	C	0.423928	0.794741	-0.17908
16	C	1.791442	1.008823	-0.24734
17	C	2.748981	-0.00679	-0.22994
18	O	-5.09002	-3.5538	0.079402
19	C	-7.39721	-1.8672	0.098847
20	O	-8.42368	-1.17866	0.108941
21	H	-7.49004	-2.96316	0.136938
22	C	2.541551	-1.50353	-0.04575
23	C	3.962778	-1.99881	-0.02313
24	C	4.824869	-0.92131	-0.19475
25	N	4.062897	0.244651	-0.35109
26	C	1.855422	-1.82232	1.288368
27	C	1.785837	-2.12185	-1.22975
28	C	4.663937	1.558464	-0.51373
29	C	4.960139	2.224015	0.822949
30	C	5.576379	3.599928	0.62316
31	C	4.469651	-3.27402	0.14199
32	C	5.857204	-3.44761	0.142073
33	C	6.708711	-2.35404	-0.01967
34	C	6.204965	-1.06414	-0.19241
35	H	-2.76147	-2.36924	-0.00486
36	H	-6.68487	0.690339	-0.00985
37	H	-5.0781	2.650329	-0.13884
38	H	0.734019	3.41118	0.404892
39	H	0.046784	3.572076	-1.19976
40	H	-1.02438	5.203825	0.26897
41	H	-1.39127	3.965103	1.46921
42	H	-3.42723	4.45585	0.117283
43	H	-2.53437	4.110599	-1.35927
44	H	0.062502	-0.22444	-0.1524
45	H	2.155548	2.02823	-0.30614
46	H	0.82128	-1.47514	1.302936
47	H	1.85636	-2.90468	1.441155
48	H	2.388806	-1.35739	2.121379
49	H	0.75074	-1.77966	-1.2705
50	H	2.271857	-1.87063	-2.1758
51	H	1.783422	-3.20973	-1.12235
52	H	5.577873	1.434248	-1.09759
53	H	3.989945	2.170527	-1.11506
54	H	4.029093	2.30217	1.395077
55	H	5.635507	1.581486	1.398624
56	H	5.795028	4.079478	1.581388
57	H	6.51374	3.536022	0.060874
58	H	4.900439	4.258252	0.067568
59	H	3.805099	-4.12193	0.274287
60	H	6.276422	-4.43965	0.274633
61	H	7.783375	-2.5047	-0.00978
62	H	6.869149	-0.21582	-0.31025

Table S20. Converged atomic positions for probe BH^+ with the SMD_{SAS} method.

Row	Symbol	X	Y	Z
1	C	-5.99971	-1.32935	0.137454
2	C	-4.87989	-2.1937	0.112603
3	C	-3.58964	-1.68278	0.026584
4	C	-3.42248	-0.31015	-0.02356
5	C	-4.51089	0.585438	-0.00591
6	C	-5.78912	0.0478	0.076916
7	O	-2.15385	0.149085	-0.08331
8	C	-1.86573	1.480672	-0.13042
9	C	-2.93922	2.431362	-0.14704
10	C	-4.21939	1.983034	-0.07256
11	C	-0.52225	1.829999	-0.1602
12	C	-0.15196	3.292643	-0.16698
13	C	-1.25147	4.176802	0.41069
14	C	-2.58256	3.883775	-0.26888
15	C	0.455246	0.817648	-0.18566
16	C	1.81598	1.026786	-0.25763
17	C	2.770485	-0.00054	-0.23459
18	O	-5.10159	-3.52504	0.176162
19	C	-7.35095	-1.86234	0.231713
20	O	-8.36411	-1.17047	0.25889
21	H	-7.44214	-2.95676	0.281163
22	C	2.551259	-1.49278	-0.03659
23	C	3.968297	-1.9973	-0.01086
24	C	4.837332	-0.92853	-0.19482
25	N	4.079847	0.242258	-0.35798
26	C	1.86358	-1.7952	1.300738
27	C	1.790242	-2.11627	-1.21473
28	C	4.691201	1.551989	-0.52689
29	C	4.995529	2.214023	0.809564
30	C	5.625976	3.583291	0.608846
31	C	4.467771	-3.2738	0.168137
32	C	5.853894	-3.45377	0.168392
33	C	6.712521	-2.36758	-0.0076
34	C	6.216289	-1.0773	-0.19432
35	H	-2.72715	-2.33986	0.0086
36	H	-6.64682	0.712066	0.094867
37	H	-5.04602	2.686735	-0.07158
38	H	0.770058	3.433461	0.405014
39	H	0.075023	3.607039	-1.19487
40	H	-0.98708	5.231225	0.289687
41	H	-1.34429	3.986829	1.486782
42	H	-3.39092	4.492981	0.143691
43	H	-2.50429	4.136037	-1.33497
44	H	0.088077	-0.199	-0.15396
45	H	2.186924	2.043382	-0.31845
46	H	-4.26035	-3.99637	0.156933
47	H	0.831309	-1.44261	1.311768
48	H	1.859333	-2.87625	1.461634
49	H	2.399389	-1.32653	2.129937
50	H	0.756618	-1.77014	-1.25588
51	H	2.275389	-1.87557	-2.16381
52	H	1.783858	-3.20295	-1.09734
53	H	5.602191	1.417244	-1.11233
54	H	4.020607	2.167862	-1.12737
55	H	4.065509	2.301774	1.381877
56	H	5.664482	1.564358	1.384502
57	H	5.849692	4.060223	1.567175
58	H	6.562468	3.509309	0.046519
59	H	4.956692	4.248511	0.053505
60	H	3.799108	-4.11651	0.311529
61	H	6.267902	-4.44645	0.312085
62	H	7.786088	-2.5251	0.002284
63	H	6.884441	-0.23376	-0.32282

Table S21. Converged atomic positions for probe A-H_2O with the SMD method.

Row	Symbol	X	Y	Z
1	C	-4.86186	0.017165	0.004428
2	C	-3.92886	-1.09605	-0.08327
3	C	-2.54169	-0.77666	-0.12718
4	C	-2.12213	0.528797	-0.08821
5	C	-3.01923	1.617442	-0.00334
6	C	-4.39587	1.309827	0.03742
7	O	-0.77984	0.765011	-0.14102
8	C	-0.26068	2.016306	-0.09186
9	C	-1.11963	3.130609	0.003667
10	C	-2.48085	2.913499	0.03739
11	C	1.146732	2.107631	-0.13694
12	C	1.766129	3.484373	-0.14115
13	C	0.812639	4.549517	-0.67448
14	C	-0.51231	4.502926	0.07725
15	C	1.909252	0.949733	-0.15129
16	C	3.305741	0.896695	-0.163
17	C	4.035551	-0.28056	-0.16561
18	O	-4.33197	-2.30238	-0.12718
19	C	-6.34354	-0.1914	0.076088
20	S	-7.11307	-1.13011	-1.31114
21	C	-7.43732	-2.65961	-0.38586
22	S	-6.83779	-0.9945	1.670457
23	C	-7.95596	-2.26136	0.981254
24	C	3.521037	-1.7163	-0.1053
25	C	4.811551	-2.49497	-0.08212
26	C	5.882656	-1.60824	-0.13172
27	N	5.388378	-0.30185	-0.21204
28	C	2.730079	-1.98364	1.183803
29	C	2.703641	-2.08579	-1.35194
30	C	6.253565	0.865714	-0.20796
31	C	6.564583	1.349123	1.203331
32	C	7.467197	2.574128	1.173193
33	C	5.037478	-3.85701	-0.0162
34	C	6.358534	-4.31713	0.00653
35	C	7.421771	-3.41494	-0.03365
36	C	7.202617	-2.03763	-0.10252
37	H	-1.81275	-1.57735	-0.19623
38	H	-5.09973	2.136262	0.101585
39	H	-3.15668	3.762857	0.102189
40	H	2.078132	3.748886	0.878815
41	H	2.678312	3.469034	-0.74544
42	H	1.267845	5.539964	-0.58181
43	H	0.629618	4.374228	-1.74177
44	H	-1.22305	5.233953	-0.31919
45	H	-0.34173	4.765925	1.130169
46	H	1.365104	0.013999	-0.14415
47	H	3.858265	1.829747	-0.15953
48	H	-6.8253	0.78693	0.040923
49	H	-6.51778	-3.23926	-0.31317
50	H	-8.19092	-3.22057	-0.94569
51	H	-8.97383	-1.86912	0.920239
52	H	-7.94254	-3.10996	1.671745
53	H	2.509491	-3.05235	1.252677
54	H	1.783992	-1.43939	1.198013
55	H	3.310871	-1.69398	2.063444
56	H	1.75597	-1.5451	-1.38839
57	H	3.264362	-1.86605	-2.26426
58	H	2.484084	-3.15675	-1.33136
59	H	7.171455	0.59735	-0.73592
60	H	5.775279	1.651962	-0.79578
61	H	5.624113	1.579004	1.716881
62	H	7.041999	0.534993	1.760522
63	H	7.696315	2.921508	2.184598
64	H	8.416257	2.353933	0.672994
65	H	6.991785	3.401387	0.635635
66	H	4.206451	-4.55534	0.020442
67	H	6.558132	-5.38271	0.059852
68	H	8.441838	-3.78591	-0.00846
69	H	8.034292	-1.34228	-0.12564
70	O	-2.61189	-4.30876	-0.29299
71	H	-1.74102	-3.93051	-0.44423
72	H	-3.2188	-3.52329	-0.2327

Table S22. Converged atomic positions for probe AH^+-H_2O with the SMD method.

Row	Symbol	X	Y	Z
1	C	-4.89663	0.255085	-0.1585
2	C	-3.97613	-0.81851	-0.02451
3	C	-2.60795	-0.56692	0.063623
4	C	-2.16088	0.739869	0.018097
5	C	-3.03434	1.82825	-0.11397
6	C	-4.40625	1.547535	-0.20177
7	O	-0.81994	0.938129	0.111541
8	C	-0.27072	2.177517	0.064634
9	C	-1.11698	3.31779	-0.07759
10	C	-2.46678	3.131597	-0.15551
11	C	1.121595	2.250093	0.153668
12	C	1.773401	3.610207	0.204624
13	C	0.81314	4.700909	0.667455
14	C	-0.46659	4.667845	-0.15968
15	C	1.870978	1.069651	0.164162
16	C	3.254211	1.003196	0.220183
17	C	3.976803	-0.18935	0.189115
18	O	-4.45611	-2.06249	0.004964
19	C	-6.38124	0.056201	-0.24506
20	S	-6.98163	-1.01011	-1.62613
21	C	-7.50211	-2.42312	-0.60678
22	S	-7.06409	-0.59584	1.345924
23	C	-8.16451	-1.86562	0.636612
24	C	3.454243	-1.6104	0.015641
25	C	4.738162	-2.39771	-0.00672
26	C	5.810916	-1.5273	0.148546
27	N	5.316709	-0.22218	0.291496
28	C	2.586891	-2.05244	1.204367
29	C	2.712933	-1.78056	-1.3188
30	C	6.190522	0.935374	0.413715
31	C	6.581752	1.503291	-0.94493
32	C	7.497053	2.708633	-0.78564
33	C	4.959662	-3.75462	-0.15324
34	C	6.278244	-4.21978	-0.14896
35	C	7.344074	-3.33111	-0.00139
36	C	7.129177	-1.96049	0.150427
37	H	-1.89814	-1.37993	0.166249
38	H	-5.09789	2.378577	-0.30798
39	H	-3.12677	3.988482	-0.26077
40	H	2.636434	3.568936	0.876633
41	H	2.17019	3.862262	-0.78837
42	H	1.292343	5.680633	0.58599
43	H	0.56554	4.546513	1.724729
44	H	-1.17792	5.43244	0.164244
45	H	-0.22436	4.880471	-1.20979
46	H	1.311411	0.144611	0.111344
47	H	3.817497	1.927758	0.276741
48	H	-3.72935	-2.73132	0.094223
49	H	-6.83755	1.029171	-0.4301
50	H	-6.63973	-3.04264	-0.36079
51	H	-8.21103	-3.00189	-1.2052
52	H	-8.29033	-2.63919	1.399862
53	H	-9.13989	-1.43113	0.406027
54	H	1.641511	-1.50843	1.240733
55	H	2.3636	-3.11787	1.103149
56	H	3.113979	-1.89827	2.149442
57	H	1.778502	-1.21689	-1.33557
58	H	3.334594	-1.44772	-2.154
59	H	2.478205	-2.83852	-1.46273
60	H	5.684344	1.685025	1.024692
61	H	7.074155	0.620669	0.972764
62	H	7.077997	0.719857	-1.52869
63	H	5.672657	1.781247	-1.48997
64	H	7.783501	3.117097	-1.7588
65	H	7.004479	3.506498	-0.22003
66	H	8.415273	2.440167	-0.25275
67	H	4.128646	-4.44336	-0.27235
68	H	6.475639	-5.28075	-0.26519
69	H	8.361959	-3.70833	-0.00636
70	H	7.96165	-1.27426	0.257895
71	O	-2.65508	-4.00497	0.232445
72	H	-2.64807	-4.35534	1.130501
73	H	-1.74544	-3.72917	0.0713

Table S23. Converged atomic positions for probe B-H_2O with the SMD method.

Row	Symbol	X	Y	Z
1	C	-6.01298	-1.33094	0.155366
2	C	-4.89299	-2.24377	0.306938
3	C	-3.58886	-1.66955	0.246701
4	C	-3.43327	-0.3216	0.061186
5	C	-4.52469	0.576695	-0.08285
6	C	-5.80483	0.031362	-0.03148
7	O	-2.15831	0.1586	0.023346
8	C	-1.88317	1.477738	-0.15933
9	C	-2.94739	2.409772	-0.31484
10	C	-4.23508	1.951398	-0.26881
11	C	-0.53153	1.831192	-0.19315
12	C	-0.16824	3.293134	-0.28964
13	C	-1.29866	4.208737	0.168392
14	C	-2.59482	3.850359	-0.54845
15	C	0.443321	0.828944	-0.16909
16	C	1.810392	1.049256	-0.23551
17	C	2.767679	0.035829	-0.23449
18	O	-5.06002	-3.49524	0.486536
19	C	-7.3624	-1.85437	0.200112
20	O	-8.38656	-1.1804	0.07841
21	H	-7.44692	-2.94149	0.354974
22	C	2.558093	-1.46996	-0.13328
23	C	3.979702	-1.96844	-0.14572
24	C	4.844103	-0.8838	-0.24343
25	N	4.084822	0.293114	-0.32099
26	C	1.881589	-1.86137	1.189783
27	C	1.793043	-2.02169	-1.34564
28	C	4.69215	1.615435	-0.36212
29	C	4.916153	2.18411	1.033548
30	C	5.550059	3.565765	0.960723
31	C	4.483116	-3.25403	-0.07124
32	C	5.870099	-3.42988	-0.08952
33	C	6.723871	-2.32963	-0.17862
34	C	6.224179	-1.02883	-0.25689
35	H	-2.71642	-2.30537	0.35305
36	H	-6.66335	0.688412	-0.14071
37	H	-5.06124	2.648391	-0.38381
38	H	0.725295	3.481106	0.314164
39	H	0.106237	3.532765	-1.32623
40	H	-1.03406	5.252935	-0.02134
41	H	-1.44161	4.100712	1.250512
42	H	-3.42487	4.48325	-0.22235
43	H	-2.46997	4.018206	-1.6269
44	H	0.084757	-0.19056	-0.11261
45	H	2.16835	2.070729	-0.29277
46	H	0.844687	-1.52341	1.230631
47	H	1.889111	-2.95049	1.283782
48	H	2.419781	-1.43753	2.041549
49	H	0.758978	-1.67292	-1.36326
50	H	2.276991	-1.72337	-2.27923
51	H	1.785045	-3.11368	-1.29392
52	H	5.636868	1.523965	-0.90153
53	H	4.053584	2.270741	-0.95747
54	H	3.954975	2.230925	1.557871
55	H	5.5562	1.497376	1.598851
56	H	5.714024	3.977294	1.960554
57	H	6.518643	3.530299	0.451036
58	H	4.910272	4.265008	0.412166
59	H	3.816004	-4.10765	0.002657
60	H	6.287022	-4.43026	-0.03023
61	H	7.798443	-2.48363	-0.18557
62	H	6.893387	-0.1782	-0.32017
63	O	-2.96542	-5.13012	0.714451
64	H	-2.16878	-4.59703	0.639539
65	H	-3.70939	-4.48168	0.624865

Table S24. Converged atomic positions for probe BH^+-H_2O with the SMD method.

Row	Symbol	X	Y	Z
1	C	-5.97293	-1.33821	0.150837
2	C	-4.85164	-2.20597	0.236483
3	C	-3.5603	-1.68011	0.169866
4	C	-3.40409	-0.31537	0.024868
5	C	-4.49314	0.575852	-0.06484
6	C	-5.77136	0.031986	0.000729
7	O	-2.13349	0.148993	-0.02309
8	C	-1.8507	1.474868	-0.16345
9	C	-2.92381	2.416226	-0.28098
10	C	-4.20566	1.96586	-0.22179
11	C	-0.50758	1.826765	-0.1929
12	C	-0.13792	3.288768	-0.24872
13	C	-1.26522	4.19656	0.231214
14	C	-2.56368	3.857162	-0.49067
15	C	0.471322	0.817335	-0.19289
16	C	1.831045	1.041456	-0.25077
17	C	2.793842	0.02497	-0.2662
18	O	-5.07341	-3.51407	0.380747
19	C	-7.32822	-1.87296	0.218723
20	O	-8.33983	-1.18557	0.147602
21	H	-7.41644	-2.96307	0.341619
22	C	2.58575	-1.48136	-0.19781
23	C	4.007712	-1.97651	-0.21569
24	C	4.869927	-0.88867	-0.28309
25	N	4.105105	0.288641	-0.33589
26	C	1.906444	-1.89852	1.116583
27	C	1.823671	-2.00928	-1.42271
28	C	4.710539	1.614276	-0.3389
29	C	4.923053	2.142172	1.074404
30	C	5.555584	3.525924	1.045172
31	C	4.51454	-3.2623	-0.16847
32	C	5.901787	-3.43205	-0.18338
33	C	6.753331	-2.32758	-0.24213
34	C	6.250375	-1.02721	-0.29292
35	H	-2.68784	-2.32024	0.232129
36	H	-6.63436	0.687196	-0.0657
37	H	-5.03378	2.664238	-0.30412
38	H	0.753916	3.455606	0.363575
39	H	0.142061	3.553526	-1.27749
40	H	-0.99918	5.243761	0.062389
41	H	-1.40594	4.066424	1.311045
42	H	-3.39131	4.489451	-0.15851
43	H	-2.43579	4.034517	-1.56716
44	H	0.111465	-0.20264	-0.1605
45	H	2.189019	2.063726	-0.28405
46	H	-4.22348	-4.03077	0.433942
47	H	0.868967	-1.56291	1.16036
48	H	1.916015	-2.98921	1.188381
49	H	2.441894	-1.49063	1.977718
50	H	0.788409	-1.66408	-1.4334
51	H	2.307475	-1.68978	-2.34922
52	H	1.819958	-3.102	-1.39304
53	H	5.658647	1.537848	-0.87412
54	H	4.074502	2.28365	-0.92049
55	H	3.957573	2.172723	1.591972
56	H	5.559695	1.440236	1.624519
57	H	5.711514	3.908402	2.057698
58	H	6.527915	3.505916	0.541976
59	H	4.918795	4.239771	0.512274
60	H	3.850109	-4.11961	-0.11823
61	H	6.321907	-4.43211	-0.14544
62	H	7.828215	-2.47871	-0.24701
63	H	6.916569	-0.17292	-0.33307
64	O	-2.88478	-4.97887	0.511574
65	H	-2.2093	-4.53826	1.03987
66	H	-3.05472	-5.80884	0.972307

Table S25. Converged atomic positions for probe A-H_2O with the SMD_{Bondi} method.

Row	Symbol	X	Y	Z
1	C	-4.8626	0.023569	0.021632
2	C	-3.93027	-1.0931	-0.0223
3	C	-2.54175	-0.77739	-0.05063
4	C	-2.1221	0.528205	-0.0348
5	C	-3.01964	1.619776	0.015242
6	C	-4.39756	1.315825	0.038253
7	O	-0.77967	0.763529	-0.07633
8	C	-0.26077	2.014936	-0.0535
9	C	-1.11867	3.130945	0.014237
10	C	-2.48052	2.91485	0.038088
11	C	1.147384	2.104376	-0.09998
12	C	1.767039	3.480574	-0.12703
13	C	0.81622	4.536638	-0.68231
14	C	-0.51163	4.504203	0.064423
15	C	1.908759	0.946618	-0.10441
16	C	3.305495	0.892505	-0.12612
17	C	4.040022	-0.28071	-0.13851
18	O	-4.34221	-2.29515	-0.04376
19	C	-6.34363	-0.19298	0.053578
20	S	-7.0553	-1.16012	-1.349
21	C	-7.4401	-2.66759	-0.40932
22	S	-6.88234	-0.98297	1.640113
23	C	-8.00242	-2.23545	0.929681
24	C	3.533309	-1.71915	-0.08077
25	C	4.827983	-2.4919	-0.07668
26	C	5.893997	-1.59958	-0.13569
27	N	5.392498	-0.29541	-0.20059
28	C	2.757133	-1.9983	1.214529
29	C	2.708143	-2.0829	-1.32413
30	C	6.250758	0.876466	-0.21179
31	C	6.590299	1.36447	1.191489
32	C	7.492564	2.588696	1.139407
33	C	5.061645	-3.8529	-0.02219
34	C	6.384654	-4.30693	-0.02077
35	C	7.442537	-3.3998	-0.07113
36	C	7.215933	-2.02349	-0.12858
37	H	-1.8128	-1.57962	-0.08914
38	H	-5.1004	2.144466	0.071129
39	H	-3.15625	3.765285	0.078998
40	H	2.077472	3.763534	0.888245
41	H	2.679955	3.455452	-0.72956
42	H	1.271796	5.528082	-0.60604
43	H	0.637594	4.343129	-1.74697
44	H	-1.21938	5.228319	-0.34907
45	H	-0.34609	4.788165	1.112484
46	H	1.362253	0.012569	-0.0841
47	H	3.856234	1.826319	-0.12905
48	H	-6.83502	0.7783	-0.01063
49	H	-6.53414	-3.26151	-0.29164
50	H	-8.18065	-3.2243	-0.98933
51	H	-9.00714	-1.82052	0.823579
52	H	-8.03447	-3.06943	1.636156
53	H	2.535233	-3.06679	1.278208
54	H	1.811844	-1.45428	1.249482
55	H	3.348478	-1.71767	2.089688
56	H	1.76566	-1.53377	-1.36226
57	H	3.268165	-1.86968	-2.23811
58	H	2.477806	-3.1514	-1.30344
59	H	7.15857	0.61419	-0.75938
60	H	5.757625	1.659486	-0.79168
61	H	5.661922	1.596421	1.725049
62	H	7.078693	0.552822	1.742211
63	H	7.740836	2.939715	2.144705
64	H	8.432032	2.366553	0.622757
65	H	7.008346	3.414667	0.608263
66	H	4.235708	-4.55642	0.021431
67	H	6.589687	-5.37154	0.02379
68	H	8.464334	-3.76584	-0.06298
69	H	8.045298	-1.32618	-0.15921
70	O	-2.69942	-4.35815	-0.21964
71	H	-1.94209	-4.0767	-0.73911
72	H	-3.2784	-3.55246	-0.16135

Table S26. Converged atomic positions for probe AH^{+}-H_2O with the SMD_{Bondi} method.

Row	Symbol	X	Y	Z	Row	Symbol	X	Y	Z
1	C	-4.89625	0.256224	-0.13085	37	H	-1.88086	-1.37576	0.011359
2	C	-3.96815	-0.81772	-0.08807	38	H	-5.11089	2.383334	-0.16183
3	C	-2.59877	-0.56472	-0.02617	39	H	-3.14634	3.99739	-0.11424
4	C	-2.15978	0.745764	-0.0116	40	H	2.639386	3.571093	0.882077
5	C	-3.04105	1.834341	-0.06415	41	H	2.137398	3.913164	-0.76298
6	C	-4.41375	1.551661	-0.12245	42	H	1.281289	5.68076	0.690985
7	O	-0.81865	0.947548	0.059832	43	H	0.58241	4.505756	1.803524
8	C	-0.2764	2.190282	0.066337	44	H	-1.19582	5.434768	0.313264
9	C	-1.12986	3.331151	-0.01323	45	H	-0.26894	4.942815	-1.09966
10	C	-2.48007	3.140893	-0.06365	46	H	1.308071	0.16269	0.095714
11	C	1.116263	2.265826	0.149868	47	H	3.816421	1.942875	0.223643
12	C	1.762954	3.626874	0.229207	48	H	-3.72235	-2.73329	-0.09242
13	C	0.80764	4.696827	0.747503	49	H	-6.8562	1.02134	-0.28557
14	C	-0.48814	4.687076	-0.05435	50	H	-6.65365	-3.05062	-0.45022
15	C	1.867878	1.087397	0.146621	51	H	-8.25048	-2.95089	-1.23936
16	C	3.251096	1.018736	0.19256	52	H	-8.24179	-2.73146	1.386248
17	C	3.971447	-0.17501	0.191924	53	H	-9.10966	-1.46248	0.489487
18	O	-4.45009	-2.06252	-0.11496	54	H	1.655337	-1.40578	1.357308
19	C	-6.38078	0.047087	-0.17308	55	H	2.34325	-3.03338	1.282869
20	S	-7.01963	-0.94917	-1.59026	56	H	3.13762	-1.78012	2.252251
21	C	-7.51829	-2.41244	-0.63234	57	H	1.745254	-1.28685	-1.2518
22	S	-6.99473	-0.70364	1.402118	58	H	3.291841	-1.55579	-2.07208
23	C	-8.13314	-1.91999	0.66164	59	H	2.448374	-2.90815	-1.29747
24	C	3.440893	-1.60094	0.101531	60	H	5.703416	1.732337	0.898723
25	C	4.72134	-2.39451	0.106124	61	H	7.082713	0.658131	0.880519
26	C	5.799488	-1.52176	0.196057	62	H	7.045435	0.629657	-1.62712
27	N	5.312629	-0.20821	0.270284	63	H	5.658477	1.713141	-1.61716
28	C	2.589342	-1.96933	1.326143	64	H	7.780405	3.002559	-1.9794
29	C	2.682471	-1.84396	-1.21207	65	H	7.0377	3.473267	-0.44645
30	C	6.193558	0.949088	0.317957	66	H	8.430736	2.384322	-0.45694
31	C	6.570615	1.446265	-1.0722	67	H	4.1007	-4.44917	-0.03388
32	C	7.506591	2.642735	-0.984	68	H	6.442613	-5.29566	-0.0102
33	C	4.935433	-3.75837	0.034187	69	H	8.338443	-3.72082	0.133423
34	C	6.251523	-4.22913	0.047571	70	H	7.953583	-1.27639	0.261676
35	C	7.322591	-3.33908	0.130023	71	O	-2.59558	-3.96784	-0.08382
36	C	7.115484	-1.96131	0.205544	72	H	-2.05799	-3.98473	0.715518
					73	H	-1.9619	-3.90111	-0.80644

Table S27. Converged atomic positions for probe B-H_2O with the SMD_{Bondi} method.

Row	Symbol	X	Y	Z
1	C	-6.00046	-1.34485	0.092155
2	C	-4.87708	-2.2609	0.207579
3	C	-3.57444	-1.67755	0.170229
4	C	-3.42473	-0.3237	0.033112
5	C	-4.51997	0.575024	-0.08028
6	C	-5.79896	0.021723	-0.0459
7	O	-2.15222	0.164431	0.012261
8	C	-1.88328	1.488314	-0.13078
9	C	-2.94895	2.41981	-0.25561
10	C	-4.23585	1.953619	-0.22157
11	C	-0.53056	1.846592	-0.1571
12	C	-0.17389	3.310993	-0.23081
13	C	-1.30053	4.211052	0.265742
14	C	-2.60515	3.869508	-0.44354
15	C	0.444904	0.848543	-0.14315
16	C	1.81365	1.064961	-0.21806
17	C	2.764094	0.047235	-0.21076
18	O	-5.0444	-3.51475	0.336048
19	C	-7.35053	-1.87436	0.122393
20	O	-8.37478	-1.19953	0.037866
21	H	-7.42598	-2.96837	0.230445
22	C	2.543896	-1.45461	-0.06653
23	C	3.961802	-1.96475	-0.08184
24	C	4.833007	-0.89046	-0.22304
25	N	4.082901	0.289803	-0.3245
26	C	1.880102	-1.80476	1.273892
27	C	1.762517	-2.036	-1.25481
28	C	4.698737	1.603589	-0.42878
29	C	4.976258	2.224428	0.934944
30	C	5.613028	3.598276	0.784743
31	C	4.456585	-3.25131	0.023182
32	C	5.841521	-3.43933	-0.00856
33	C	6.702157	-2.34959	-0.14118
34	C	6.211296	-1.04798	-0.25051
35	H	-2.69805	-2.31059	0.253473
36	H	-6.66214	0.676049	-0.13098
37	H	-5.06435	2.650887	-0.31211
38	H	0.728756	3.491804	0.360958
39	H	0.081031	3.574106	-1.26659
40	H	-1.04097	5.260643	0.101555
41	H	-1.42859	4.07395	1.346248
42	H	-3.43185	4.487712	-0.08295
43	H	-2.49869	4.076754	-1.51688
44	H	0.087546	-0.17125	-0.08774
45	H	2.173037	2.085089	-0.28892
46	H	0.849952	-1.44836	1.324942
47	H	1.869409	-2.89103	1.394329
48	H	2.436873	-1.37165	2.108483
49	H	0.727407	-1.69044	-1.27186
50	H	2.233613	-1.76262	-2.20211
51	H	1.753685	-3.12615	-1.17666
52	H	5.623565	1.487585	-0.99716
53	H	4.047124	2.242167	-1.02794
54	H	4.036773	2.297637	1.493804
55	H	5.633666	1.557826	1.503854
56	H	5.817986	4.046455	1.760676
57	H	6.560842	3.53811	0.239978
58	H	4.956102	4.280559	0.235567
59	H	3.785504	-4.09788	0.13056
60	H	6.251194	-4.44065	0.074185
61	H	7.775049	-2.51203	-0.15792
62	H	6.887022	-0.20596	-0.34558
63	O	-3.05081	-5.26246	0.538588
64	H	-2.21924	-4.78335	0.499173
65	H	-3.7509	-4.56535	0.460303

Table S28. Converged atomic positions for probe BH^{+}-H_2O with the SMD_{Bondi} method.

Row	Symbol	X	Y	Z
1	C	-5.96627	-1.35386	0.079403
2	C	-4.84222	-2.21647	0.166077
3	C	-3.55255	-1.68396	0.133679
4	C	-3.40106	-0.31585	0.020433
5	C	-4.49319	0.571336	-0.06804
6	C	-5.76972	0.019218	-0.03645
7	O	-2.13269	0.156311	0.002084
8	C	-1.85502	1.484259	-0.11373
9	C	-2.93001	2.423388	-0.2221
10	C	-4.21022	1.965048	-0.18891
11	C	-0.51219	1.841056	-0.13194
12	C	-0.14779	3.304253	-0.17297
13	C	-1.27595	4.19993	0.327002
14	C	-2.57583	3.870835	-0.39673
15	C	0.467951	0.835303	-0.13635
16	C	1.827932	1.055386	-0.20975
17	C	2.783137	0.032594	-0.22427
18	O	-5.05952	-3.52948	0.278002
19	C	-7.32225	-1.89574	0.113731
20	O	-8.33322	-1.21095	0.047013
21	H	-7.40498	-2.98969	0.206418
22	C	2.563291	-1.47006	-0.1072
23	C	3.980653	-1.97846	-0.13982
24	C	4.849939	-0.90148	-0.26319
25	N	4.094525	0.280534	-0.33591
26	C	1.907032	-1.8432	1.23121
27	C	1.775219	-2.02936	-1.30204
28	C	4.710352	1.597697	-0.41987
29	C	4.997916	2.189156	0.954707
30	C	5.637182	3.564056	0.826452
31	C	4.477992	-3.26633	-0.0633
32	C	5.862703	-3.44926	-0.10568
33	C	6.721525	-2.35555	-0.22049
34	C	6.228191	-1.05338	-0.3012
35	H	-2.68042	-2.32409	0.195862
36	H	-6.63656	0.668835	-0.10393
37	H	-5.04014	2.661135	-0.26605
38	H	0.747398	3.467765	0.434861
39	H	0.123301	3.584273	-1.19993
40	H	-1.01369	5.250739	0.177231
41	H	-1.41203	4.050071	1.40466
42	H	-3.4036	4.492064	-0.04513
43	H	-2.45509	4.076413	-1.46875
44	H	0.109352	-0.18467	-0.09758
45	H	2.188015	2.076222	-0.26085
46	H	-4.20652	-4.03226	0.351442
47	H	0.876642	-1.4895	1.293022
48	H	1.899154	-2.93134	1.332103
49	H	2.467664	-1.42363	2.070007
50	H	0.738169	-1.68966	-1.30356
51	H	2.23776	-1.73415	-2.24687
52	H	1.773064	-3.12073	-1.24556
53	H	5.630324	1.490743	-0.9972
54	H	4.054628	2.247402	-1.00148
55	H	4.062112	2.253691	1.520696
56	H	5.657466	1.510178	1.506005
57	H	5.849042	3.992182	1.809812
58	H	6.581492	3.511889	0.274969
59	H	4.978559	4.258361	0.29479
60	H	3.809352	-4.11632	0.029893
61	H	6.275115	-4.45104	-0.04554
62	H	7.794387	-2.51616	-0.24605
63	H	6.901469	-0.20818	-0.3833
64	O	-2.79198	-4.87279	0.471529
65	H	-2.46191	-4.9388	1.374149
66	H	-2.84463	-5.78153	0.15618

Table S29. Converged atomic positions for probe A-H_2O with the SMD_{SAS} method.

Row	Symbol	X	Y	Z
1	C	-4.84124	0.000521	0.019212
2	C	-3.90777	-1.10786	-0.01134
3	C	-2.52468	-0.7946	-0.02593
4	C	-2.10502	0.514343	-0.01313
5	C	-3.00277	1.601708	0.02252
6	C	-4.3778	1.296651	0.034617
7	O	-0.76329	0.748307	-0.0448
8	C	-0.24351	1.999413	-0.03093
9	C	-1.10625	3.119077	0.028167
10	C	-2.46386	2.902797	0.042891
11	C	1.161161	2.091656	-0.08379
12	C	1.780507	3.468513	-0.11594
13	C	0.829084	4.523789	-0.66952
14	C	-0.49753	4.490879	0.078228
15	C	1.927286	0.933135	-0.09677
16	C	3.320773	0.880407	-0.13242
17	C	4.059735	-0.29366	-0.15723
18	O	-4.31745	-2.32441	-0.03257
19	C	-6.32268	-0.21444	0.038807
20	S	-7.02015	-1.17254	-1.38579
21	C	-7.55179	-2.63991	-0.45101
22	S	-6.87464	-1.02357	1.609151
23	C	-8.09583	-2.16232	0.879631
24	C	3.557503	-1.73107	-0.0956
25	C	4.851529	-2.50067	-0.10636
26	C	5.915918	-1.60574	-0.16798
27	N	5.409132	-0.30272	-0.22988
28	C	2.799038	-2.00844	1.208772
29	C	2.714881	-2.09605	-1.3243
30	C	6.256547	0.877056	-0.22606
31	C	6.535348	1.381405	1.183412
32	C	7.389712	2.639117	1.156454
33	C	5.08742	-3.86142	-0.06043
34	C	6.4111	-4.31425	-0.06806
35	C	7.467915	-3.40531	-0.11798
36	C	7.238337	-2.02894	-0.16704
37	H	-1.79324	-1.59457	-0.05473
38	H	-5.0837	2.122569	0.057749
39	H	-3.14159	3.751682	0.074454
40	H	2.093848	3.754096	0.897918
41	H	2.692667	3.442105	-0.71983
42	H	1.284202	5.515848	-0.59403
43	H	0.649278	4.329628	-1.73397
44	H	-1.20487	5.217711	-0.33115
45	H	-0.32786	4.770978	1.126778
46	H	1.385781	-0.00291	-0.0741
47	H	3.872207	1.81355	-0.13663
48	H	-6.81249	0.75689	-0.02112
49	H	-6.71302	-3.32454	-0.31953
50	H	-8.3283	-3.129	-1.044
51	H	-9.05583	-1.65636	0.758125
52	H	-8.21559	-2.99106	1.581747
53	H	2.579001	-3.07715	1.276886
54	H	1.856087	-1.46119	1.250594
55	H	3.401238	-1.72612	2.076108
56	H	1.772464	-1.54653	-1.34427
57	H	3.260342	-1.88083	-2.24676
58	H	2.488686	-3.16552	-1.30012
59	H	7.18653	0.622263	-0.73672
60	H	5.775287	1.650454	-0.82842
61	H	5.581642	1.57881	1.685304
62	H	7.03673	0.589945	1.751921
63	H	7.597417	3.000531	2.167586
64	H	8.350638	2.454455	0.664932
65	H	6.887862	3.445049	0.610867
66	H	4.261546	-4.56463	-0.01534
67	H	6.617701	-5.37887	-0.03104
68	H	8.490251	-3.76993	-0.11629
69	H	8.064978	-1.32882	-0.19881
70	O	-2.64609	-4.35763	-0.13255
71	H	-1.76699	-4.0209	-0.32579
72	H	-3.229	-3.54926	-0.09894

Table S30. Converged atomic positions for probe AH^+-H_2O with the SMD_{SAS} method.

Row	Symbol	X	Y	Z
1	C	-4.89658	0.236372	-0.11238
2	C	-3.96827	-0.83649	-0.08909
3	C	-2.59838	-0.5852	-0.057
4	C	-2.15741	0.725354	-0.0454
5	C	-3.03944	1.813958	-0.07316
6	C	-4.41248	1.532496	-0.10624
7	O	-0.81556	0.929317	-0.00105
8	C	-0.2741	2.173191	0.0106
9	C	-1.13047	3.314675	-0.04305
10	C	-2.48026	3.122318	-0.0706
11	C	1.119205	2.249051	0.087683
12	C	1.763794	3.611586	0.168878
13	C	0.814619	4.676914	0.705931
14	C	-0.49151	4.671875	-0.07784
15	C	1.874312	1.071652	0.089255
16	C	3.256452	1.003297	0.158721
17	C	3.982969	-0.18768	0.176888
18	O	-4.44652	-2.08966	-0.10455
19	C	-6.38228	0.035876	-0.13127
20	S	-7.04893	-0.94768	-1.55055
21	C	-7.62435	-2.38395	-0.59261
22	S	-6.98285	-0.72484	1.443706
23	C	-8.20007	-1.86393	0.70815
24	C	3.463694	-1.61415	0.059074
25	C	4.746149	-2.40092	0.088701
26	C	5.817225	-1.52288	0.212467
27	N	5.32114	-0.21383	0.294509
28	C	2.579452	-2.00145	1.251912
29	C	2.741349	-1.84326	-1.27475
30	C	6.188946	0.951248	0.368449
31	C	6.57595	1.466482	-1.01059
32	C	7.484482	2.68126	-0.90351
33	C	4.968924	-3.76281	0.010903
34	C	6.287034	-4.22829	0.049315
35	C	7.351625	-3.33361	0.161765
36	C	7.134891	-1.9575	0.24462
37	H	-1.8837	-1.3992	-0.03675
38	H	-5.1097	2.364499	-0.12792
39	H	-3.14907	3.977674	-0.09928
40	H	2.650447	3.554135	0.807511
41	H	2.123727	3.906611	-0.8264
42	H	1.286739	5.662135	0.650877
43	H	0.602952	4.477143	1.763342
44	H	-1.19481	5.415455	0.306558
45	H	-0.28531	4.938766	-1.12316
46	H	1.319169	0.145214	0.030459
47	H	3.820905	1.92723	0.201896
48	H	-3.7102	-2.74841	-0.08095
49	H	-6.85411	1.012602	-0.22979
50	H	-6.79994	-3.07679	-0.41957
51	H	-8.39017	-2.87793	-1.19516
52	H	-8.35246	-2.67122	1.428563
53	H	-9.14795	-1.34697	0.545408
54	H	1.64543	-1.43789	1.261147
55	H	2.339246	-3.06593	1.186425
56	H	3.100105	-1.82416	2.196257
57	H	1.804903	-1.28577	-1.32603
58	H	3.371166	-1.54087	-2.11529
59	H	2.514713	-2.90732	-1.38083
60	H	5.682899	1.725099	0.948209
61	H	7.075361	0.667587	0.938054
62	H	7.075236	0.66381	-1.56463
63	H	5.664525	1.717507	-1.56415
64	H	7.765912	3.054083	-1.89233
65	H	6.990508	3.498217	-0.36737
66	H	8.406112	2.439626	-0.36392
67	H	4.138407	-4.45552	-0.08226
68	H	6.484571	-5.29347	-0.01246
69	H	8.369213	-3.71025	0.18361
70	H	7.966059	-1.26707	0.326685
71	O	-2.54436	-3.97298	-0.04396
72	H	-2.04967	-3.91045	0.780755
73	H	-1.88947	-3.81091	-0.73232

Table S31. Converged atomic positions for probe B-H_2O with the SMD_{SAS} method.

Row	Symbol	X	Y	Z
1	C	-6.00983	-1.34556	0.049407
2	C	-4.88854	-2.26489	0.046461
3	C	-3.58765	-1.69029	-0.00811
4	C	-3.43059	-0.3285	-0.05229
5	C	-4.5241	0.576807	-0.05308
6	C	-5.80112	0.030712	0.00074
7	O	-2.15821	0.150285	-0.08943
8	C	-1.88191	1.480616	-0.14183
9	C	-2.95046	2.424098	-0.16502
10	C	-4.23514	1.965155	-0.10922
11	C	-0.5316	1.835519	-0.17137
12	C	-0.17137	3.300875	-0.18382
13	C	-1.27245	4.177016	0.403688
14	C	-2.60492	3.881638	-0.27211
15	C	0.444449	0.831881	-0.19457
16	C	1.811367	1.042371	-0.26746
17	C	2.76386	0.021104	-0.23905
18	O	-5.05119	-3.5356	0.091207
19	C	-7.35516	-1.86765	0.108664
20	O	-8.38396	-1.1845	0.119206
21	H	-7.44171	-2.96384	0.146926
22	C	2.547589	-1.4725	-0.03769
23	C	3.966113	-1.97467	-0.0072
24	C	4.834313	-0.90439	-0.19172
25	N	4.078421	0.264029	-0.3616
26	C	1.857971	-1.77363	1.298821
27	C	1.790154	-2.10172	-1.2149
28	C	4.685912	1.573842	-0.53393
29	C	4.970363	2.253574	0.798282
30	C	5.587879	3.627595	0.589724
31	C	4.4663	-3.25005	0.175879
32	C	5.85288	-3.43032	0.180225
33	C	6.710501	-2.34375	0.004313
34	C	6.213524	-1.0539	-0.18646
35	H	-2.71465	-2.33356	-0.01136
36	H	-6.65996	0.695656	0.003172
37	H	-5.06275	2.668699	-0.11534
38	H	0.755724	3.449538	0.377999
39	H	0.04163	3.617511	-1.21422
40	H	-1.01273	5.233477	0.288758
41	H	-1.36003	3.979386	1.478992
42	H	-3.41426	4.479339	0.155993
43	H	-2.53674	4.153794	-1.33416
44	H	0.081353	-0.18635	-0.16162
45	H	2.179135	2.059959	-0.33495
46	H	0.825047	-1.42267	1.307531
47	H	1.854679	-2.85426	1.463233
48	H	2.391333	-1.30184	2.12792
49	H	0.755996	-1.75733	-1.25999
50	H	2.276874	-1.86349	-2.16388
51	H	1.784365	-3.18821	-1.09473
52	H	5.60556	1.439022	-1.10601
53	H	4.020441	2.180901	-1.14972
54	H	4.034441	2.337276	1.361575
55	H	5.640966	1.617121	1.386188
56	H	5.799334	4.116251	1.544966
57	H	6.52938	3.558563	0.035049
58	H	4.915886	4.280422	0.02299
59	H	3.797485	-4.09279	0.319261
60	H	6.266848	-4.42256	0.327091
61	H	7.78432	-2.49995	0.01743
62	H	6.881945	-0.21046	-0.3147
63	O	-3.00479	-5.1751	0.441011
64	H	-2.19063	-4.67433	0.341243
65	H	-3.73267	-4.51269	0.296131

Table S32. Converged atomic positions for probe BH^+-H_2O with the SMD_{SAS} method.

Row	Symbol	X	Y	Z
1	C	-5.96694	-1.35188	0.14126
2	C	-4.84231	-2.21669	0.119527
3	C	-3.55599	-1.68799	0.017573
4	C	-3.40313	-0.31596	-0.04732
5	C	-4.49645	0.574378	-0.03044
6	C	-5.76937	0.02623	0.065052
7	O	-2.13712	0.15215	-0.11775
8	C	-1.8575	1.484087	-0.16915
9	C	-2.93537	2.428287	-0.18628
10	C	-4.21337	1.972292	-0.10643
11	C	-0.51483	1.840182	-0.19863
12	C	-0.15105	3.304439	-0.19516
13	C	-1.25949	4.182735	0.374669
14	C	-2.58536	3.881946	-0.31244
15	C	0.464917	0.83161	-0.23284
16	C	1.827069	1.042913	-0.27562
17	C	2.781411	0.016225	-0.2659
18	O	-5.05077	-3.53792	0.201098
19	C	-7.31248	-1.89268	0.24963
20	O	-8.33293	-1.20961	0.275639
21	H	-7.39408	-2.98722	0.31137
22	C	2.561272	-1.48392	-0.14152
23	C	3.978654	-1.98714	-0.09551
24	C	4.849017	-0.90961	-0.20861
25	N	4.093981	0.26552	-0.34376
26	C	1.833179	-1.85243	1.157258
27	C	1.840335	-2.04863	-1.37346
28	C	4.706806	1.580538	-0.45704
29	C	4.986505	2.200409	0.904495
30	C	5.625315	3.57252	0.756265
31	C	4.476196	-3.26956	0.041045
32	C	5.862403	-3.4479	0.07154
33	C	6.722105	-2.35348	-0.03302
34	C	6.227919	-1.05676	-0.17608
35	H	-2.68355	-2.33003	-0.00128
36	H	-6.63269	0.683464	0.082247
37	H	-5.0438	2.671695	-0.10635
38	H	0.764837	3.447057	0.386397
39	H	0.085188	3.623383	-1.21958
40	H	-0.99999	5.238489	0.254471
41	H	-1.35827	3.993287	1.450324
42	H	-3.39859	4.489148	0.093524
43	H	-2.50172	4.131273	-1.37884
44	H	0.099539	-0.18598	-0.23178
45	H	2.198575	2.060832	-0.29595
46	H	-4.19347	-4.0352	0.204501
47	H	0.795709	-1.51529	1.149934
48	H	1.839332	-2.93948	1.2716
49	H	2.334893	-1.41289	2.022877
50	H	0.810153	-1.69457	-1.43438
51	H	2.36026	-1.76512	-2.29187
52	H	1.825801	-3.13964	-1.30826
53	H	5.628285	1.466453	-1.03097
54	H	4.045575	2.214978	-1.0485
55	H	4.045428	2.275253	1.46042
56	H	5.641561	1.531583	1.473497
57	H	5.828321	4.022533	1.732052
58	H	6.574315	3.510822	0.213631
59	H	4.97099	4.254969	0.203893
60	H	3.805784	-4.11859	0.128081
61	H	6.274847	-4.44539	0.182558
62	H	7.795459	-2.50959	-0.00053
63	H	6.897304	-0.20752	-0.24936
64	O	-2.79883	-4.95653	0.216323
65	H	-2.17844	-4.62023	0.872731
66	H	-2.98263	-5.86184	0.490827

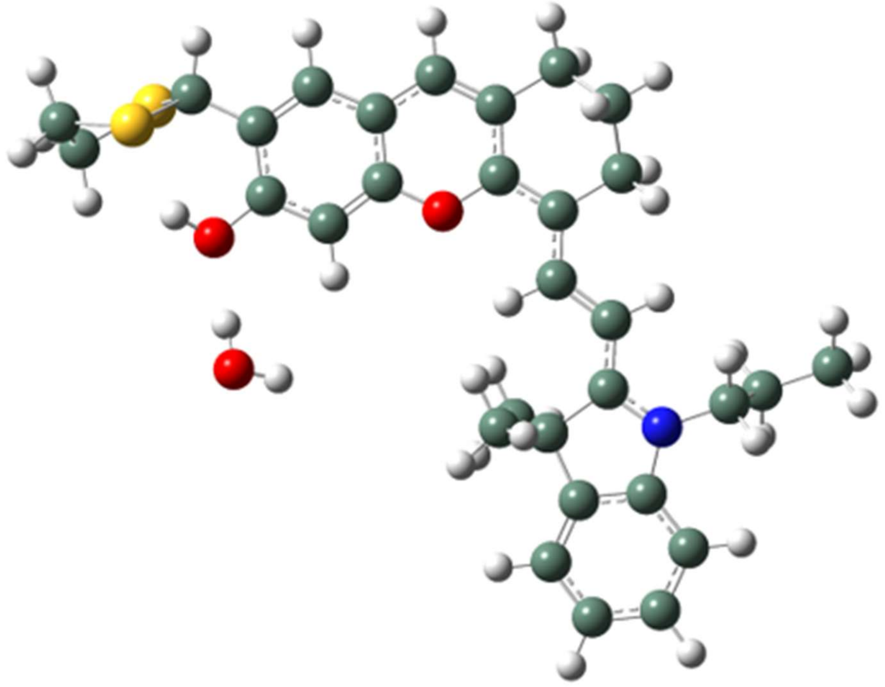

Figure S13. Drawing of the model with the intramolecular H···S bond preserved.

Table S33. Converged atomic positions for probe A-H_2O with the intramolecular H···S bond preserved and with the SMD method.

Row	Symbol	X	Y	Z	Row	Symbol	X	Y	Z
1	C	-4.89504	0.241978	0.011649	37	H	-1.89327	-1.40621	0.113305
2	C	-3.96403	-0.82542	0.055523	38	H	-5.10988	2.369958	-0.03661
3	C	-2.59841	-0.58386	0.069212	39	H	-3.14474	3.981069	-0.05927
4	C	-2.15572	0.726789	0.045467	40	H	2.651013	3.559638	0.82895
5	C	-3.03836	1.814759	0.013894	41	H	2.134966	3.884758	-0.81549
6	C	-4.41133	1.539131	0.000133	42	H	1.292795	5.671846	0.619302
7	O	-0.81283	0.925582	0.07364	43	H	0.60753	4.51345	1.759353
8	C	-0.26931	2.170143	0.062543	44	H	-1.18853	5.42236	0.270274
9	C	-1.12805	3.312931	-0.01021	45	H	-0.27683	4.895255	-1.14209
10	C	-2.47661	3.125269	-0.01852	46	H	1.321683	0.14232	0.07996
11	C	1.120289	2.249829	0.12377	47	H	3.818642	1.940196	0.189086
12	C	1.768792	3.611209	0.183481	48	H	-5.26858	-2.17114	0.445717
13	C	0.820149	4.688741	0.697817	49	H	-6.88147	0.981054	-0.00601
14	C	-0.48525	4.665989	-0.08821	50	H	-6.91723	-3.02072	-1.06397
15	C	1.877608	1.070106	0.120133	51	H	-8.43146	-2.5028	-1.83689
16	C	3.258654	1.012587	0.157961	52	H	-8.53137	-2.8455	0.768109
17	C	3.987881	-0.17946	0.149326	53	H	-9.14448	-1.28673	0.162504
18	O	-4.36328	-2.12287	0.066507	54	H	1.664077	-1.46959	1.261128
19	C	-6.37449	0.020315	-0.09515	55	H	2.392321	-3.07974	1.174107
20	S	-6.89595	-0.68427	-1.73761	56	H	3.144226	-1.82175	2.172901
21	C	-7.6585	-2.21991	-1.11737	57	H	1.781461	-1.26282	-1.32251
22	S	-6.98901	-1.09478	1.238797	58	H	3.332601	-1.51385	-2.14507
23	C	-8.26297	-1.92674	0.239668	59	H	2.486948	-2.88475	-1.40252
24	C	3.468825	-1.60635	0.028059	60	H	5.699067	1.731293	0.897051
25	C	4.754965	-2.38951	0.022066	61	H	7.090244	0.668285	0.863787
26	C	5.82566	-1.51071	0.135726	62	H	7.058281	0.676646	-1.63986
27	N	5.326628	-0.20232	0.235648	63	H	5.652644	1.738502	-1.61874
28	C	2.611499	-2.01063	1.237386	64	H	7.758118	3.06474	-1.9652
29	C	2.718399	-1.82181	-1.29499	65	H	7.000944	3.508128	-0.43011
30	C	6.198679	0.961788	0.30611	66	H	8.412245	2.441966	-0.44532
31	C	6.569885	1.480429	-1.07755	67	H	4.151426	-4.44588	-0.16373
32	C	7.486239	2.691018	-0.9741	68	H	6.501886	-5.27297	-0.14601
33	C	4.980594	-3.75015	-0.07722	69	H	8.383984	-3.68547	0.03887
34	C	6.30099	-4.20923	-0.06735	70	H	7.976142	-1.24482	0.217003
35	C	7.364773	-3.31194	0.038566	71	O	-2.51786	-4.06134	1.03475
36	C	7.145721	-1.93773	0.142047	72	H	-3.16077	-3.40971	0.711635
					73	H	-1.67075	-3.72542	0.725973

Table S34. Converged atomic positions for probe A-H_2O with the intramolecular H···S bond preserved and with the SMD_{Bondi} method.

Row	Symbol	X	Y	Z
1	C	-4.87536	0.185724	-0.01548
2	C	-3.93648	-0.87591	0.027192
3	C	-2.57283	-0.62357	0.057622
4	C	-2.14009	0.690369	0.059586
5	C	-3.03056	1.771996	0.039162
6	C	-4.401	1.485893	0.001226
7	O	-0.79954	0.898974	0.100675
8	C	-0.26531	2.146516	0.11081
9	C	-1.13124	3.284089	0.063168
10	C	-2.47838	3.08612	0.040878
11	C	1.124755	2.233596	0.165726
12	C	1.764114	3.597597	0.253055
13	C	0.810993	4.65474	0.800117
14	C	-0.49861	4.643717	0.021289
15	C	1.888235	1.059714	0.129582
16	C	3.270036	1.006234	0.148976
17	C	4.004754	-0.18095	0.116491
18	O	-4.32359	-2.17673	0.026008
19	C	-6.34911	-0.04557	-0.16363
20	S	-6.78893	-0.67942	-1.85827
21	C	-7.67404	-2.18799	-1.33937
22	S	-7.00062	-1.22612	1.087874
23	C	-8.29102	-1.92793	0.018487
24	C	3.489611	-1.61083	0.010719
25	C	4.77932	-2.38834	-0.02023
26	C	5.847893	-1.50404	0.067463
27	N	5.345169	-0.19788	0.172549
28	C	2.664339	-2.00938	1.244218
29	C	2.711366	-1.84013	-1.29376
30	C	6.214152	0.968555	0.230307
31	C	6.57013	1.492059	-1.15567
32	C	7.498235	2.693911	-1.05806
33	C	5.009657	-3.74785	-0.12064
34	C	6.331786	-4.20072	-0.13799
35	C	7.392916	-3.2985	-0.05719
36	C	7.169548	-1.92533	0.047124
37	H	-1.85883	-1.43805	0.086911
38	H	-5.10362	2.312806	-0.03258
39	H	-3.15254	3.937212	0.013731
40	H	2.650557	3.53835	0.891988
41	H	2.121947	3.898406	-0.74089
42	H	1.276331	5.642664	0.745501
43	H	0.606252	4.450885	1.857878
44	H	-1.20445	5.3844	0.405945
45	H	-0.29903	4.906742	-1.02612
46	H	1.336279	0.130505	0.074238
47	H	3.826502	1.935458	0.184785
48	H	-5.2533	-2.22685	0.345784
49	H	-6.87289	0.902628	-0.04752
50	H	-6.98618	-3.03546	-1.3103
51	H	-8.44321	-2.38085	-2.09109
52	H	-8.62847	-2.85242	0.493106
53	H	-9.12846	-1.23003	-0.043
54	H	1.724315	-1.45798	1.301651
55	H	2.430359	-3.07542	1.187076
56	H	3.227117	-1.82889	2.163322
57	H	1.768376	-1.29167	-1.30863
58	H	3.303439	-1.53225	-2.15898
59	H	2.485927	-2.90497	-1.39198
60	H	5.722228	1.736318	0.829617
61	H	7.11222	0.676744	0.778031
62	H	7.044064	0.688872	-1.73051
63	H	5.649294	1.761263	-1.68457
64	H	7.7562	3.073193	-2.05044
65	H	7.030277	3.511256	-0.49993
66	H	8.4311	2.433257	-0.54778
67	H	4.183229	-4.44856	-0.18727
68	H	6.535671	-5.26337	-0.21862
69	H	8.413383	-3.66704	-0.078
70	H	7.999469	-1.23037	0.10127
71	O	-2.72011	-3.81667	1.724877
72	H	-3.28311	-3.30112	1.126549
73	H	-2.04752	-3.19043	2.007123

Table S35. Converged atomic positions for probe A-H_2O with the intramolecular H···S bond preserved and with the SMD_{SAS} method.

Row	Symbol	X	Y	Z	Row	Symbol	X	Y	Z
1	C	-4.88056	0.206705	-0.0301	37	H	-1.86949	-1.42826	-0.03656
2	C	-3.94413	-0.85674	-0.03821	38	H	-5.10183	2.334657	-0.00294
3	C	-2.57922	-0.61019	-0.03415	39	H	-3.14594	3.952508	0.013582
4	C	-2.14152	0.702518	-0.01403	40	H	2.663851	3.532647	0.828243
5	C	-3.02911	1.786908	0.000947	41	H	2.119227	3.911988	-0.79381
6	C	-4.40143	1.505512	-0.00566	42	H	1.291995	5.639754	0.720488
7	O	-0.80019	0.909188	0.007412	43	H	0.625753	4.434436	1.821783
8	C	-0.26216	2.154871	0.039993	44	H	-1.19168	5.390984	0.398105
9	C	-1.12566	3.295532	0.019599	45	H	-0.29662	4.934587	-1.04759
10	C	-2.47337	3.099853	0.014333	46	H	1.337438	0.132916	0.013297
11	C	1.128907	2.2364	0.102088	47	H	3.83288	1.923666	0.169414
12	C	1.770473	3.599042	0.199923	48	H	-5.24576	-2.21556	0.30645
13	C	0.824283	4.651949	0.76535	49	H	-6.87453	0.931445	0.026108
14	C	-0.49073	4.654401	-0.00347	50	H	-6.95236	-3.0142	-1.20429
15	C	1.889974	1.06049	0.076022	51	H	-8.47576	-2.44576	-1.92304
16	C	3.271315	0.998057	0.123646	52	H	-8.52242	-2.90243	0.667257
17	C	4.003231	-0.19187	0.115215	53	H	-9.12991	-1.3134	0.142801
18	O	-4.33751	-2.15969	-0.06339	54	H	1.687247	-1.46299	1.221144
19	C	-6.36045	-0.01851	-0.11212	55	H	2.386491	-3.08694	1.116288
20	S	-6.91198	-0.65605	-1.77474	56	H	3.159029	-1.85331	2.127301
21	C	-7.68566	-2.20594	-1.20783	57	H	1.806956	-1.27458	-1.36942
22	S	-6.95379	-1.19307	1.180188	58	H	3.361511	-1.51905	-2.18456
23	C	-8.25539	-1.96576	0.173127	59	H	2.517036	-2.89468	-1.45346
24	C	3.486611	-1.61783	-0.01304	60	H	5.70747	1.714783	0.894022
25	C	4.771696	-2.40034	-0.01285	61	H	7.104736	0.665701	0.835913
26	C	5.841562	-1.52067	0.107378	62	H	7.054808	0.70661	-1.66462
27	N	5.341506	-0.2139	0.212543	63	H	5.63536	1.747724	-1.6182
28	C	2.623288	-2.02226	1.18983	64	H	7.719551	3.107228	-1.96605
29	C	2.744402	-1.83179	-1.3384	65	H	6.968259	3.520894	-0.42065
30	C	6.205387	0.95438	0.290002	66	H	8.393059	2.475347	-0.45918
31	C	6.560002	1.495678	-1.08756	67	H	4.167824	-4.45458	-0.20125
32	C	7.459626	2.716784	-0.97816	68	H	6.517388	-5.28529	-0.17189
33	C	4.997361	-3.76045	-0.11052	69	H	8.399372	-3.69888	0.020006
34	C	6.317186	-4.22163	-0.09451	70	H	7.990903	-1.25901	0.19864
35	C	7.380541	-3.32509	0.015514	71	O	-2.69061	-3.76166	1.559969
36	C	7.160868	-1.9509	0.117936	72	H	-3.2531	-3.21274	0.986256
					73	H	-1.86039	-3.279	1.60935

Chemical Synthesis:

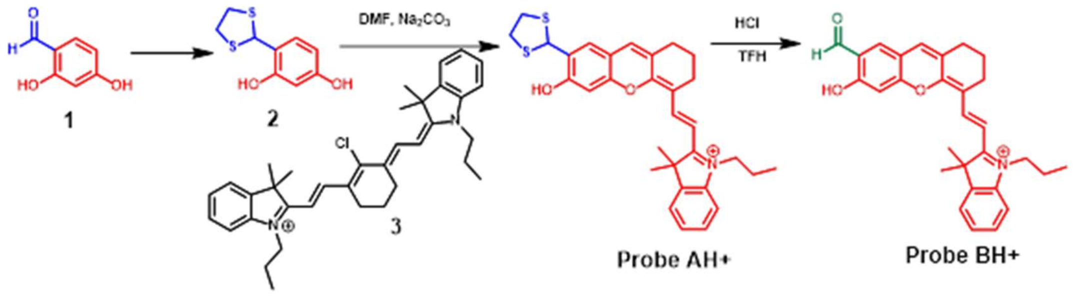

All the chemical reagents for synthesis were purchased from commercial suppliers (sigma-Adrich) and used without further purification. The solutions of cations were prepared from their chloride salts. All experiments involving buffer solution were performed with a HEPES buffer (10 mM, pH 7.4) solution unless, otherwise noted, with doubly distilled water used throughout the experiments.

Probe AH$^+$: A mixture of IR780 (1 mmol), 4-(1,3-dithiolan-2-yl)benzene-1,3-diol(2 mol) and 0.5 mL triethylamine) were added in N, N-dimethylformamide (10 mL). Then the mixture solution was stirred at 80 °C for 5 h and the DMF was removed under reduced pressure. The residue was purified by silica gel column chromatography (DCM/methanol, 50: 1, v/v) to afford probe **AH$^+$** as a blue-green solid in 50% yield. ^{1}HNMR (400 MHz, CDCl3) (400 MHz, Chloroform-d) δ 8.05 (d, J = 13.7 Hz, 1H), 7.63 (s, 1H), 7.31 (d, J = 14 Hz, 1H), 7.25 (d, J = 12Hz, 1H), 7.04 (t, J = 7.4 Hz, 1H), 6.81 (d, J = 8.0 Hz, 1H), 6.66 (s, 1H), 6.05 (s, 1H), 5.61 (d, J = 13.3 Hz, 1H), 3.74 (d, J = 8.7 Hz, 2H), 3.31 (s, 1H), 2.66 (t, J = 6.0 Hz, 1H), 2.58 (t, J = 6.3 Hz, 1H), 1.88 (p, J = 6.3 Hz, 1H), 1.80 (q, J = 7.3 Hz, 2H), 1.02 (t, J = 7.5 Hz, 2H).^{13}CNMR (100 MHz, CDCl3) δ (ppm) 172.87, 150.64, 144.36, 142.78, 140.91, 128.84, 127.76, 125.35, 122.15, 110.85, 101.69, 77.41, 77.10, 76.78, 49.25, 32.68, 28.09, 26.78, 20.69. High resolution mass spectrometer (HRMS m/z): found 516.2020 [M$^+$H], calculated 516.2025 for: $C_{31}H34NO_2S_2$

Probe BH$^+$: A mixture of Probe **AH** (1 mmol), SeO_2 (2 mol) were added in CH_3COOH (10 mL). Then the mixture solution was stirred at room temperature for 2 h and the CH_3COOH was removed under reduced pressure. The residue was purified by silica gel column chromatography (DCM/methanol, 30: 1, v/v) to afford probe **BH$^+$** as a blue-green solid with 89% yield. ^{1}HNMR (400 MHz, CDCl3) (400 MHz, Chloroform-d) δ 10.51 (s, 1H), 8.34 (d, J = 13.9 Hz, 1H), 7.77 (s, 1H), 7.40 – 7.31 (m, 2H), 7.24 – 7.15 (m, 2H), 6.99 (d, J = 8.0 Hz, 1H), 6.67 (s, 1H), 5.87 (d, J =

13.9 Hz, 1H), 3.92 (t, J = 7.5 Hz, 2H), 2.67 (t, J = 6.1 Hz, 2H), 2.60 (t, J = 6.2 Hz, 2H), 1.87 (dt, J = 15.0, 7.1 Hz, 4H), 1.61 (s, 6H), 1.05 (t, J = 7.4 Hz, 3H).^{13}C NMR (100 MHz, CDCl3) δ (ppm) 13C NMR (101 MHz, Chloroform-d) δ 192.55, 178.68, 165.10, 159.91, 157.75, 146.37, 141.41, 132.19, 130.97, 129.44, 128.14, 127.74, 122.89, 120.42, 115.21, 113.19, 106.00, 104.32, 51.41, 47.82, 28.50, 24.69, 21.89, 20.60, 11.95. High resolution mass spectrometer (HRMS, m/z): found 440.2219 [M$^+$H], calculated 440.2220 for: $C_{29}H_{30}NO_3^+$

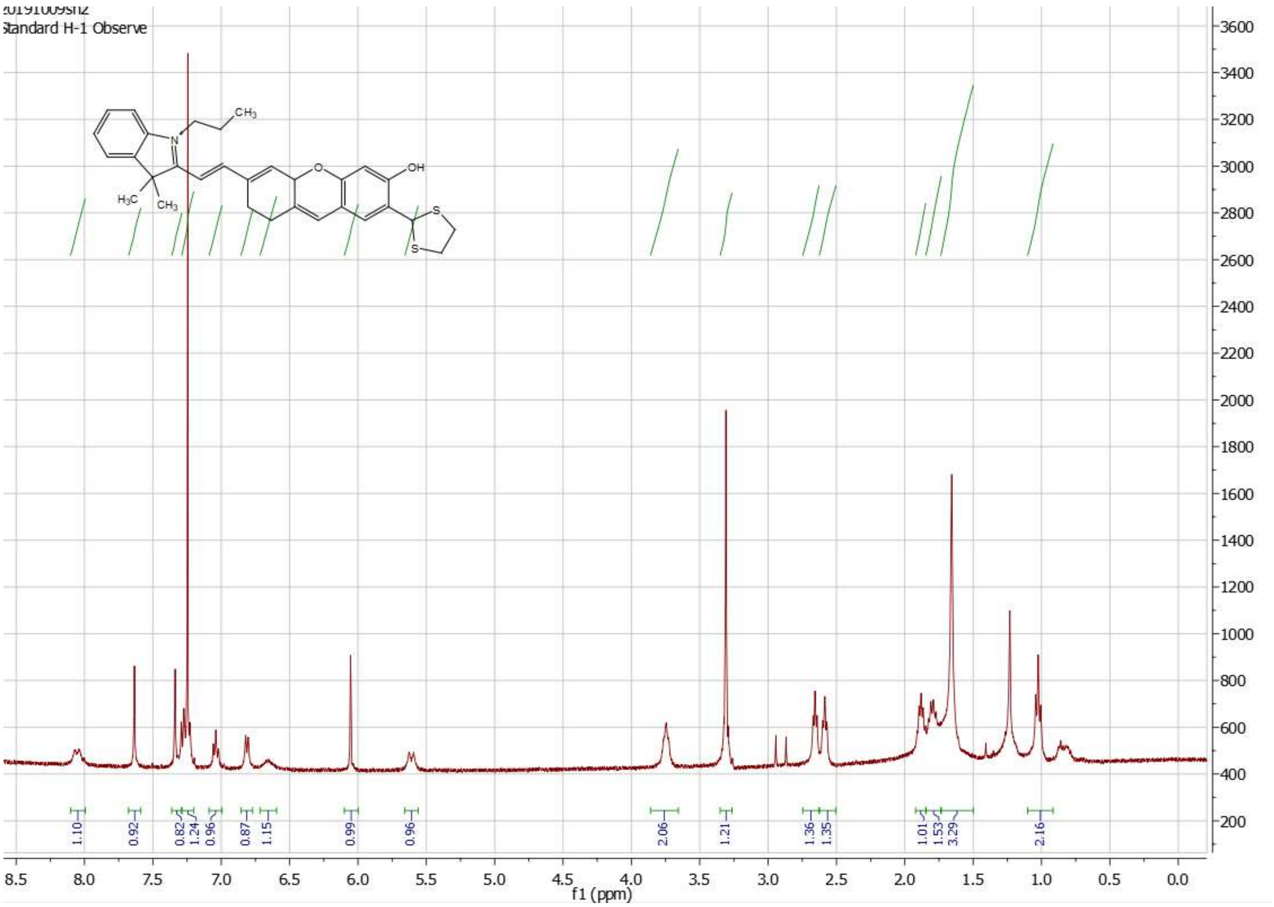

Figure S14: ^{1}H NMR spectrum of probe AH$^+$ in $CDCl_3$ solution.

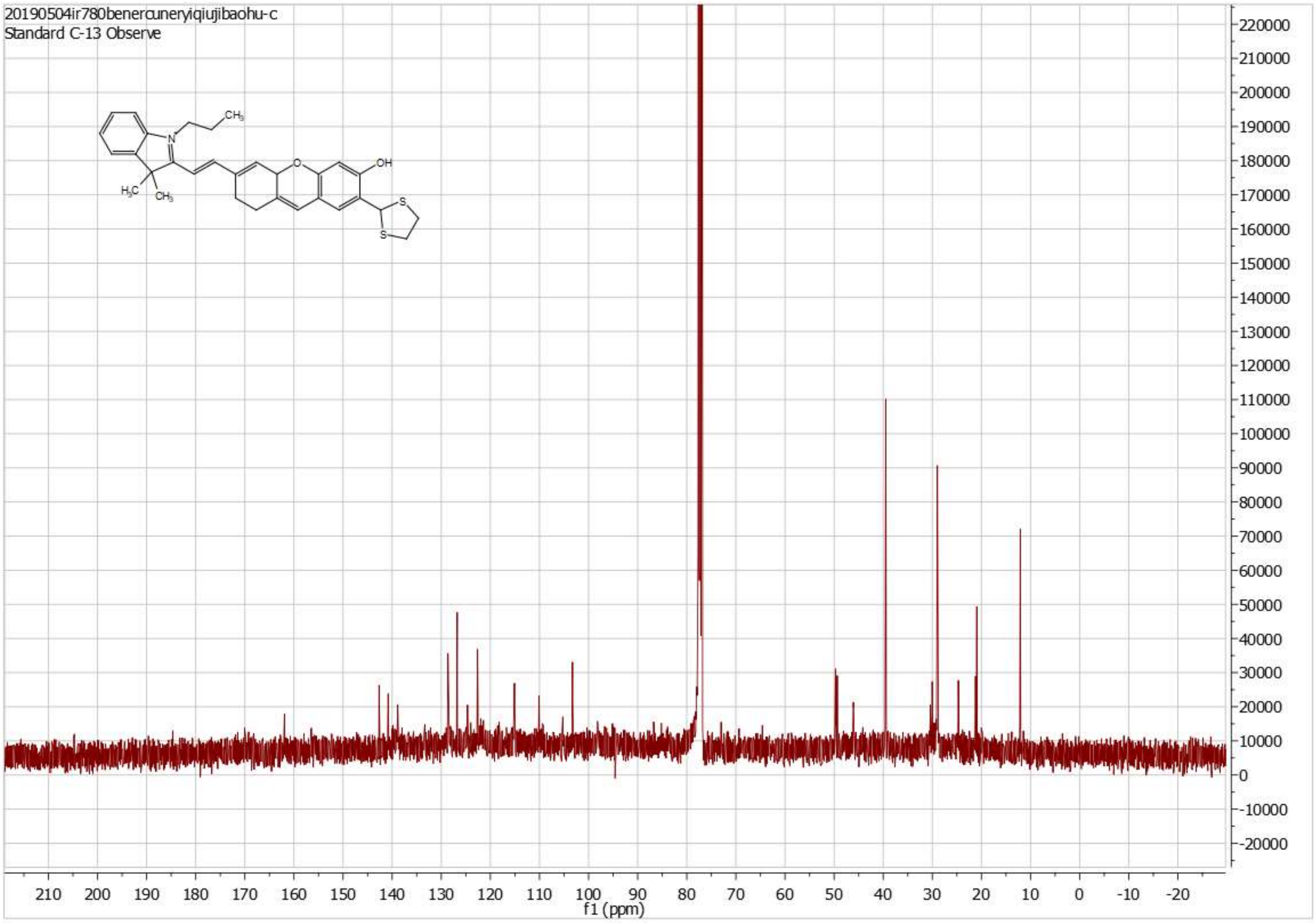

Figure S15: ^{13}C NMR spectrum of probe AH^+ in $CDCl_3$ solution.

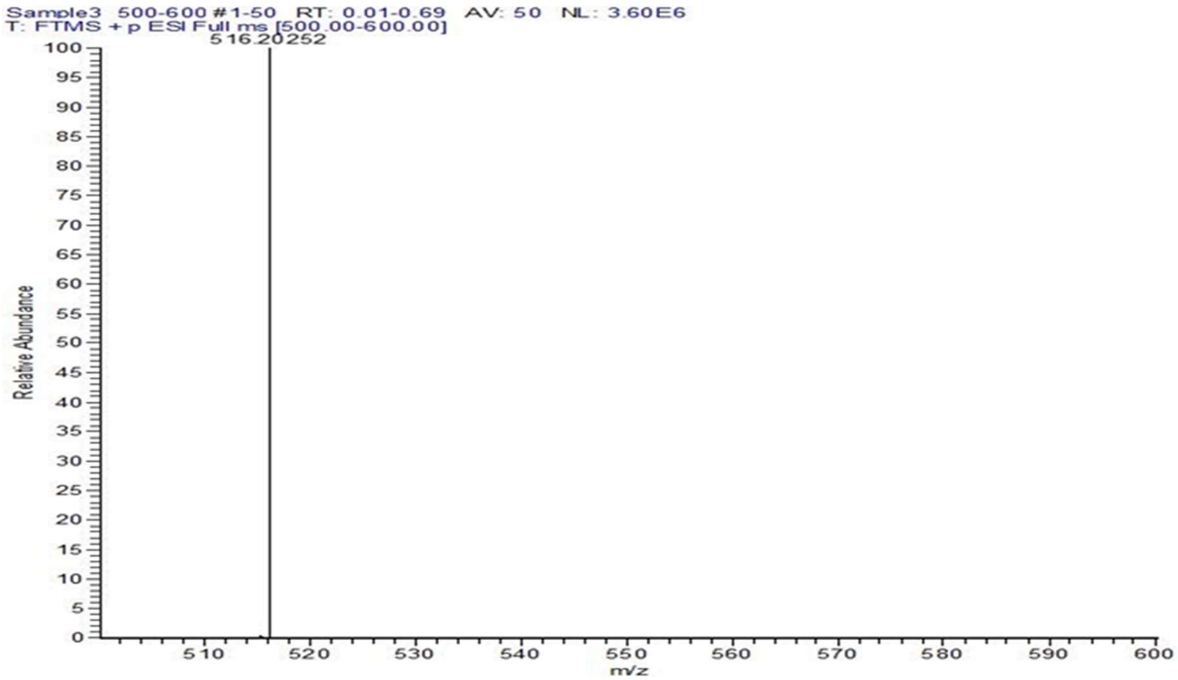

Figure S16. High-résolution mass spectrum of probe **AH**$^+$

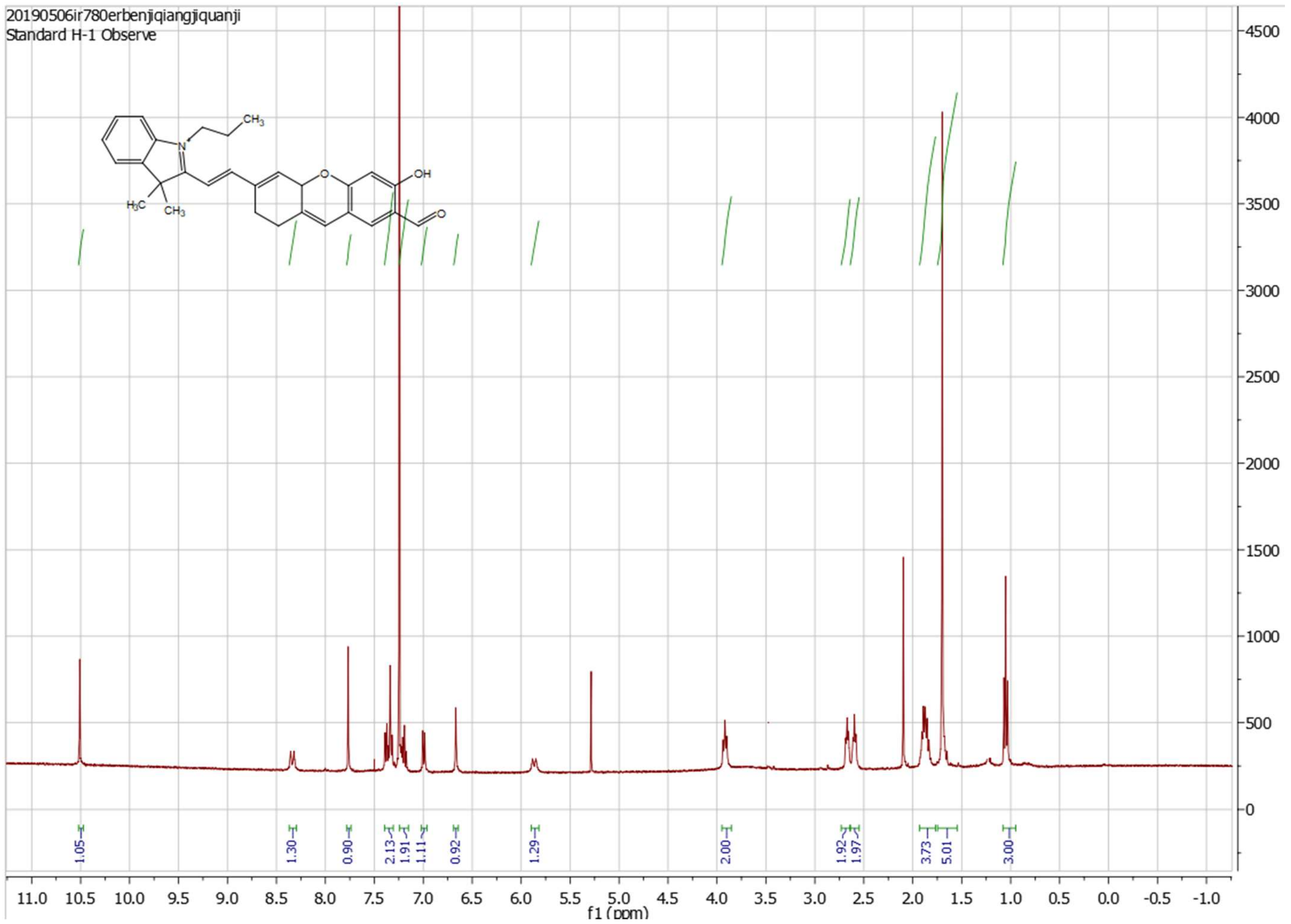

Figure S17: ^{1}H NMR spectrum of probe BH^{+} in $CDCl_3$ solution.

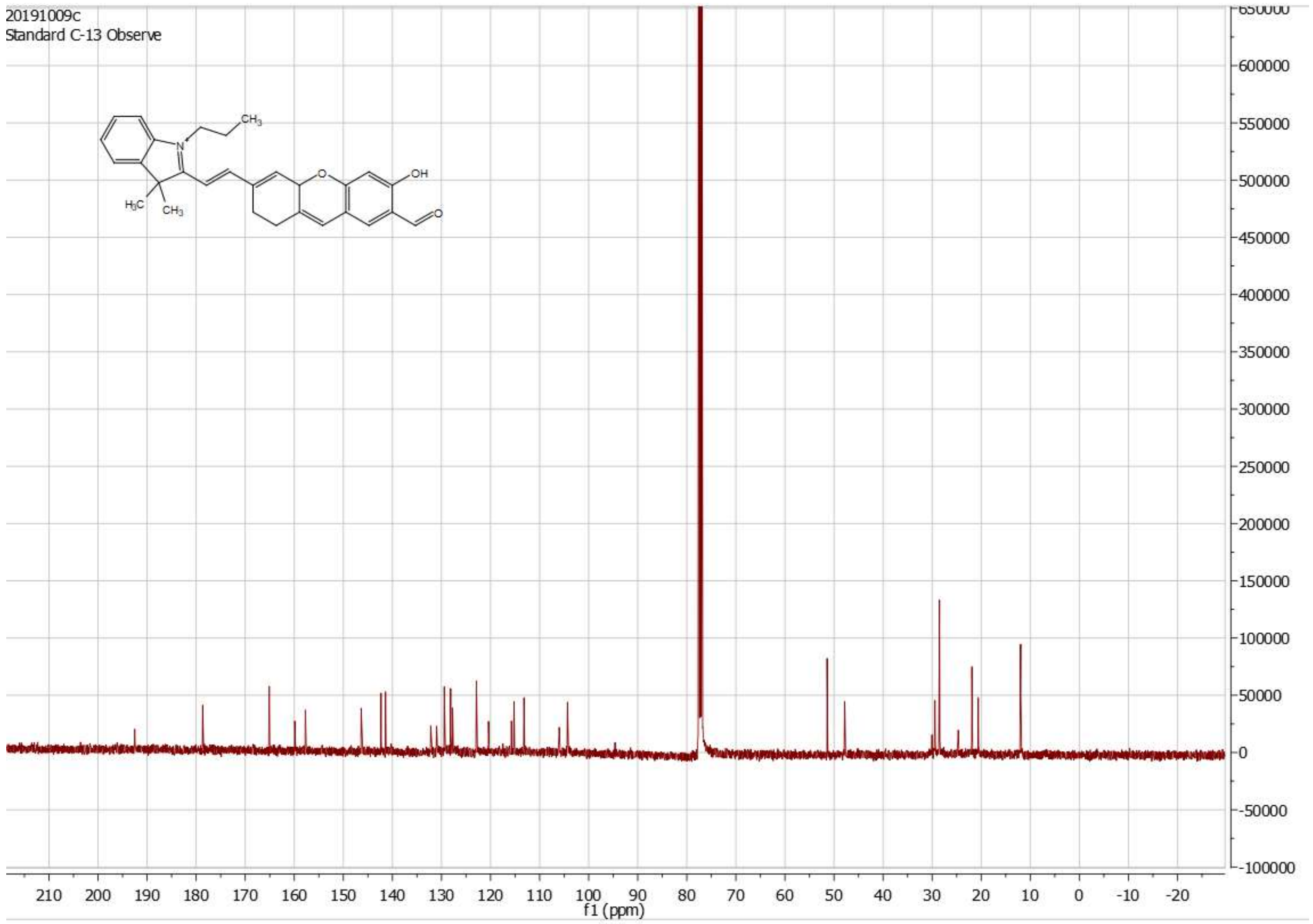

Figure S18: ^{13}C NMR spectrum of probe BH^{+} in $CDCl_3$ solution.

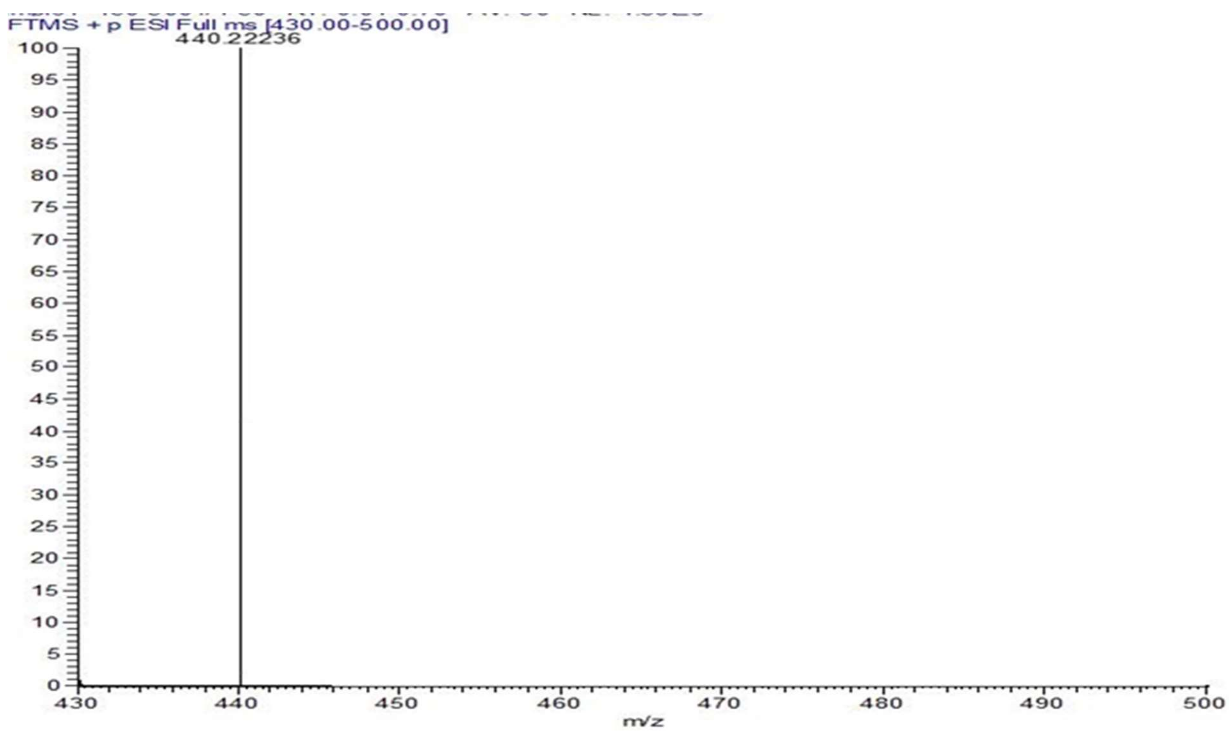

Figure S19. High-résolution mass spectrum of probe **BH**$^{+}$.

Table S36. Reversible pH cyclic data of probe **AH^+**

Cyclic N0.	$F_{718\ nm}/F_{680\ nm}$			STDEV	Average
	1st repeated	2nd repeated	3rd repeated		
1	0.3275	0.4855	0.1675	0.16	0.3268
2	13.27	12.93	12.63	0.32	12.94
3	0.5239	0.3536	0.1835	0.17	0.3537
4	12.63	13.26	12.96	0.32	12.95
5	0.5438	0.1639	0.3538	0.19	0.3538
6	13.28	12.99	12.71	0.28	12.99
7	0.5546	0.1546	0.3526	0.20	0.3539
8	12.66	12.88	13.35	0.35	12.96
9	0.5538	0.3531	0.1936	0.18	0.3668
10	13.36	12.98	12.57	0.39	12.97

Table S37. Reversible pH cyclic data of probe **BH**$^+$

Cyclic N0.	$F_{715\ nm}/F_{667\ nm}$			STDEV	Average
	1st repeated	2nd repeated	3rd repeated		
1	0.5261	0.4421	0.3621	0.082	0.4434
2	9.704	9.624	9.504	0.10	9.610
3	0.3641	0.5244	0.4432	0.081	0.4439
4	9.923	9.612	9.307	0.31	9.614
5	0.5317	0.4237	0.3127	0.11	0.4227
6	9.896	9.726	9.526	0.18	9.716
7	0.5628	0.4128	0.2928	0.14	0.4228
8	9.594	9.864	9.324	0.26	9.594
9	0.5253	0.4429	0.3637	0.081	0.4440
10	10.01	9.225	9.610	0.39	9.615

Table S38. Fluorescence intensities of 10 μM probes **AH**$^+$ in the absence and presence of different cations (200 μM) in pH 7.4 buffers under excitation at 635 nm.

Item	$F_{718\ nm}$			STDEV	Average
	1st repeated	2nd repeated	3rd repeated		
Blank	310910	305650	300210	5350	305590
K^+	297840	301580	308330	5316	302583
Mg^{2+}	306740	302590	296650	5071	301993
Al^{3+}	299820	295660	290720	4555	295400
Ba^{2+}	287840	293490	298530	5348	293287
Fe^{3+}	308630	303160	297340	5646	303043
Co^{2+}	290190	294180	300260	5070	294877
Ni^{2+}	302400	296440	292900	4801	297247
Sn^{4+}	301460	295760	289620	5921	295613
Cu^{2+}	316840	310900	305650	5598	311130
Zn^{2+}	294670	298020	304750	5134	299147
Cd^{2+}	291960	298830	303260	5694	298017
Mn^{2+}	310830	314560	320500	4877	315297
Cr^{3+}	291900	297730	303160	5631	297597
Pb^{2+}	307920	301400	296630	5668	301983
Fe^{2+}	307940	303180	297250	5356	302790

Table S39. Fluorescence intensities of 10 μM probes **BH**$^+$ in the absence and presence of different cations (200 μM) in pH 7.4 buffers under excitation at 635 nm.

Item	$F_{715\ nm}$			STDEV	Average
	1st repeated	2nd repeated	3rd repeated		
Blank	1241350	1237530	1233680	3835	1237520
K^+	1248600	1239990	1231370	8615	1239987
Mg^{2+}	1217500	1221610	1225770	4135	1221627
Al^{3+}	1255090	1247640	1240160	7465	1247630
Ba^{2+}	1276340	1269980	1263550	6395	1269957
Fe^{3+}	1223600	1187740	1151930	35835	1187757
Co^{2+}	1221480	1226230	1231010	4765	1226240
Ni^{2+}	1077180	1075150	1073160	2010	1075163
Sn^{4+}	1220900	1224690	1228400	3750	1224663
Cu^{2+}	1161510	1150700	1139980	10765	1150730
Zn^{2+}	1231950	1226100	1220040	5955	1226030
Cd^{2+}	1289710	1284790	1279850	4930	1284783
Hg^{2+}	1191060	1186350	1181700	4680	1186370
Mn^{2+}	1273050	1264000	1254960	9045	1264003
Cr^{3+}	1214160	1222650	1231180	8510	1222663
Pb^{2+}	1298300	1296310	1294360	1970	1296323
Fe^{2+}	1281500	1274330	1267160	7170	1274330

Table S40. Fluorescence intensities of 10 μM probes **AH**$^+$ in the absence and presence of different anions (200 μM) in pH 7.4 buffers under excitation at 635 nm.

Item	$F_{718\ nm}$			STDEV	Average
	1st repeated	2nd repeated	3rd repeated		
Blank	304800	309700	299800	4950	304767
Cl^-	299500	304850	309750	5127	304700
CO_3^{2-}	299860	309700	304900	4920	304820
HCO_3^-	299920	296030	304790	4389	300247
ClO_4^-	288460	298580	293520	5060	293520
SO_4^{2-}	303040	308130	297850	5140	303007
PO_4^{3-}	310500	305330	300170	5165	305333
NO_3^-	305170	310370	300060	5155	305200
SO_3^{2-}	305160	299890	310260	5185	305103
$S_2O_3^{2-}$	300120	310080	305100	4980	305100
CN^-	299570	304540	294630	4955	299580
S^{2-}	293590	298680	303680	5045	298650
ClO^-	303980	309380	298980	5201	304113

Table S41. Fluorescence intensities of 10 μM probes **BH**$^{+}$ in the absence and presence of different anions (200 μM) in pH 7.4 buffers under excitation at 635 nm.

Item	$F_{715\ nm}$			STDEV	Average
	1st repeated	2nd repeated	3rd repeated		
Blank	1227580	1224590	1221620	2980	1224597
Cl^{-}	1236590	1229910	1223260	6665	1229920
CO_3^{2-}	1260560	1253050	1245570	7495	1253060
HCO_3^{-}	1223730	1227600	1231480	3875	1227603
SO_4^{2-}	1236380	1239090	1241810	2715	1239093
SO_3^{2-}	1150550	1144540	1138120	6216	1144403
HSO_3^{-}	1168700	1162800	1156898	5901	1162799
NO_3^{-}	1284770	1278280	1271810	6480	1278287
PO_4^{3-}	1308290	1302960	1297700	5295	1302983
$S_2O_3^{2-}$	1251820	1244180	1236460	7680	1244153

Table S42. Fluorescence intensities of 10 μM probes **AH**$^+$ in the absence and presence of different Amino acids (200 μM) in pH 7.4 buffers under excitation at 635 nm.

Item	$F_{718\ nm}$			STDEV	Average
	1st repeated	2nd repeated	3rd repeated		
Blank	323170	332670	313680	9495	323173
DL-Cys	315860	297240	306570	9310	306557
DL-Hcy	303000	312240	293860	9190	303033
DL-Ala	293320	302710	283940	9385	293323
DL-Arg	289380	299040	308760	9690	299060
DL-Leu	296270	306040	286520	9760	296277
DL-Met	308040	298530	317560	9515	308043
DL-Tyr	307300	316910	297730	9590	307313
GSH	315460	325240	335120	9830	325273
Glu	326310	336040	316620	9710	326323
Gly	305410	314780	295960	9410	305383

Table S43. Fluorescence intensities of 10 μM probes **BH**$^+$ in the absence and presence of different Amino acids (200 μM) in pH 7.4 buffers under excitation at 635 nm.

Item	$F_{715\ nm}$			STDEV	Average
	1st repeated	2nd repeated	3rd repeated		
Blank	1219210	1225240	1213230	6005	1219227
DL-Cys	1243840	1236810	1229850	6995	1236833
DL-Hcy	1203010	1205060	1201120	1970	1203063
DL-Ala	1171120	1173160	1172150	1020	1172143
DL-Arg	1154860	1145840	1136820	9020	1145840
DL-Leu	1191740	1201700	1211680	9970	1201707
DL-Met	1215990	1217960	1213920	2020	1215957
DL-Tyr	1217440	1220920	1213960	3480	1217440
GSH	1251260	1248350	1245420	2920	1248343
Glu	1225590	1228540	1222520	3010	1225550
Gly	1199960	1203920	1195900	4010	1199927
